# Transcriptional profiling and physiological roles of *Aedes aegypti* spermathecal-related genes

**DOI:** 10.1186/s12864-020-6543-y

**Published:** 2020-02-10

**Authors:** Tales Vicari Pascini, Marcelo Ramalho-Ortigão, José Marcos Ribeiro, Marcelo Jacobs-Lorena, Gustavo Ferreira Martins

**Affiliations:** 1 Departamento de Biologia Geral, Universidade Federal de Viçosa, Viçosa, MG 36570-900 Brazil; 2 Division of Tropical Public Health, Department of Preventive Medicine and Biostatistics, Uniformed Services University, 4301 Jones Bridge Road, Rm A-3083, Bethesda, MD 20814 USA; 3 Section of Vector Biology, Laboratory of Malaria and Vector Research, National Institute of Allergy and Infectious Diseases, National Institutes of Health, 12735 Twinbrook Parkway, Rm 2E32D, Rockville, MD 20852 USA; 4 Department of Molecular Microbiology and Immunology, Malaria Research Institute, Johns Hopkins Bloomberg School of Public Health, Baltimore, MD 21205 USA

**Keywords:** *Ae. Aegypti*, Insect reproduction, Sperm, Spermatheca, Transcriptome

## Abstract

**Background:**

Successful mating of female mosquitoes typically occurs once, with the male sperm being stored in the female spermatheca for every subsequent oviposition event. The female spermatheca is responsible for the maintenance, nourishment, and protection of the male sperm against damage during storage. *Aedes aegypti* is a major vector of arboviruses, including Yellow Fever, Dengue, Chikungunya, and Zika. Vector control is difficult due to this mosquito high reproductive capacity.

**Results:**

Following comparative RNA-seq analyses of spermathecae obtained from virgin and inseminated females, eight transcripts were selected based on their putative roles in sperm maintenance and survival, including energy metabolism, chitin components, transcriptional regulation, hormonal signaling, enzymatic activity, antimicrobial activity, and ionic homeostasis. In situ RNA hybridization confirmed tissue-specific expression of the eight transcripts. Following RNA interference (RNAi), observed outcomes varied between targeted transcripts, affecting mosquito survival, egg morphology, fecundity, and sperm motility within the spermathecae.

**Conclusions:**

This study identified spermatheca-specific transcripts associated with sperm storage in *Ae. aegypti*. Using RNAi we characterized the role of eight spermathecal transcripts on various aspects of female fecundity and offspring survival. RNAi-induced knockdown of transcript *AeSigP-66,427,* coding for a Na^+^/Ca^2+^ protein exchanger, specifically interfered with egg production and reduced sperm motility. Our results bring new insights into the molecular basis of sperm storage and identify potential targets for *Ae. aegypti* control.

## Background

The overall ability of vectors to spread pathogens is related to their reproductive capacity. Typically, high reproductive capacity is observed in vectors considered to be highly effective in the transmission of a given pathogen [1, 2]. *Aedes aegypti* (Diptera: Culicidae) is a major disease vector responsible for the transmission of arboviruses, such as Yellow Fever, Dengue, Chikungunya, and Zika. From its pantropic distribution and its role in the transmission of such pathogens, with dengue fever alone being responsible for over 100 million cases annually with 2.5 billion people at risk, attempts to control *Ae. aegypti* is carried out across much of the tropics and subtropics [3]. Control strategies, however, are usually hampered by several factors, including high oviposition rates that confer a reproductive advantage on *Ae. aegypti* [4].

For most insects, mating is a separate event from egg fertilization. In *Ae. aegypti* and other mosquitoes, mating is a single event in which the female acquires the male sperm that can last during her entire life. Though male-derived nutrients also transferred to the female during insect mating help nourish the sperm from a few hours to a few days, ultimately it is up to the female spermatheca to maintain the viability of the male sperm [5, 6]. As median survival for *Ae. aegypti* adults is 38 days at optimal conditions [7], it can be assumed that this is also the approximate time required for sperm storage and maintenance in this mosquito. During each gonotrophic cycle, once the eggs are ready for fertilization and the environmental conditions are favorable, the sperm is released from the spermatheca to fertilize the eggs during oviposition [8, 9].

**Fig. 1:**
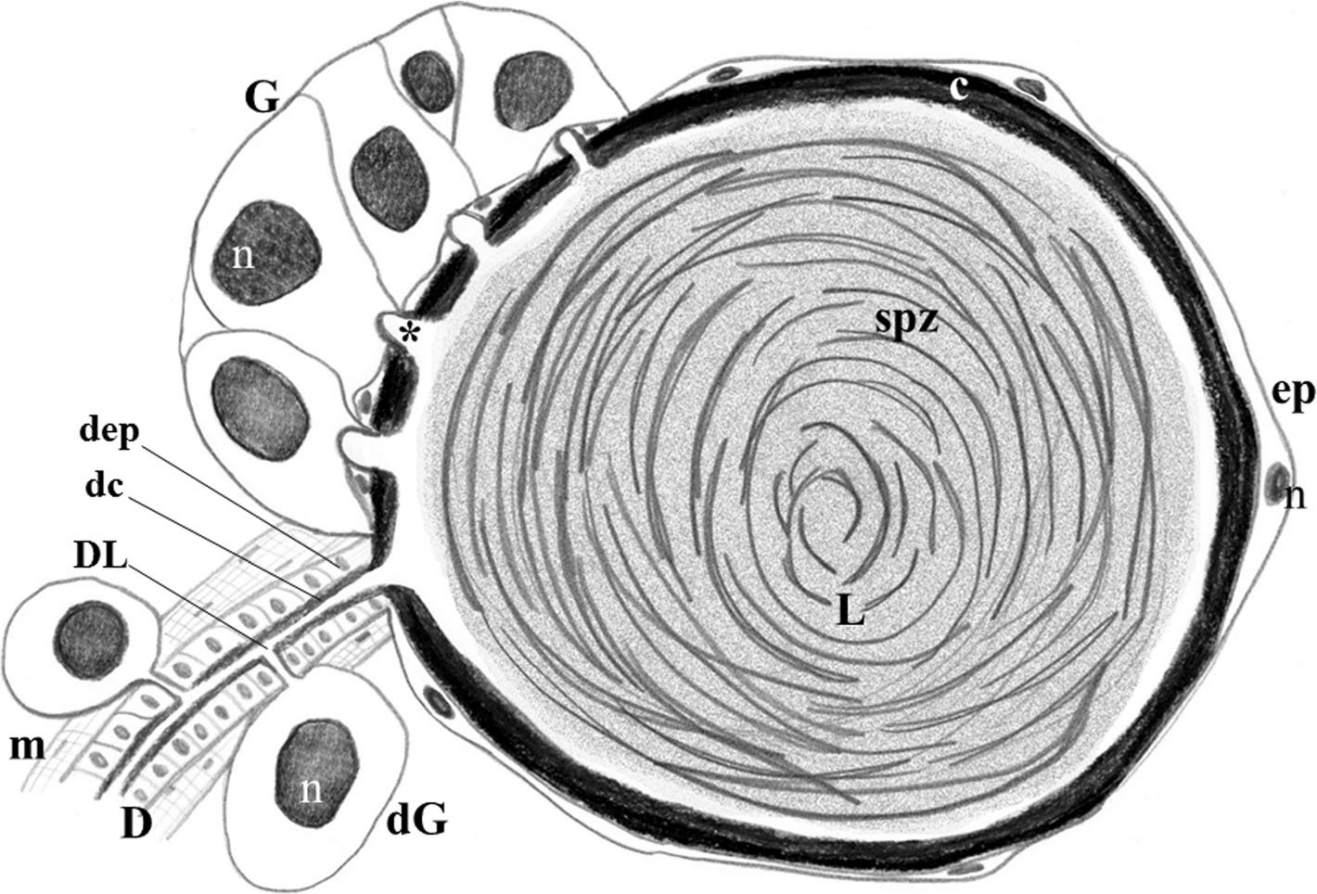
Schematic representation of a section of the *Ae. aegypti* spermatheca. (c) reservoir cuticle, (D) spermathecal duct, (dc) duct cuticle, (dep) duct epithelium with columnar cells, (dG) individual duct gland cell, (DL) duct lumen, (ep) spermathecal reservoir epithelium with flattened cells, (G) spermathecal gland with prominent cells, (L) reservoir lumen, (m) muscle, (n) nuclei, (spz) spermatozoa in circles, (*) opening of a glandular cell ductule through the cuticle of reservoir. Not to scale

In *Ae. aegypti*, there are three functional spermathecae: a large spermatheca that is centrally located, and two smaller, laterally positioned spermathecae. Both large and small spermathecae are morphologically similar with regards to cell types and gross organization [10, 11], each one comprised of a long duct (responsible for guiding the sperm migration), a rounded reservoir or capsule (for sperm storage), and a glandular portion (that produces and secretes compounds used for sperm storage and nourishment). Glandular cells (GC) present in the reservoir and in the duct form the glandular portion. Reservoir GCs form a separate unit (gland) from the flattened epithelial cells. The spermathecal gland is attached to the portion of the reservoir wall closer to the duct, while GC are individually attached to the duct. Reservoir and duct GC secrete components into the lumens of the spermathecae through cuticle interruptions or pores. The spermathecal duct is externally covered by a muscular layer, the spermathecal muscle, that is responsible for the contraction of the duct [6, 11, 12]. A general view of the morphology of *Ae. aegypti* spermatheca is depicted in Fig. 1.

Multiple factors have been linked to sperm longevity, including ions, sugars, pH, antioxidants, and enzymes for energy metabolism [13–18]. However, the current understanding of biochemical and physiological processes within the spermathecae is incomplete. In contrast, the role of components derived from the reproductive system of mosquito males (e.g., *Anopheles gambiae*) and details of sperm transfer are better understood; thanks in part to advances in male-centered transgenic approaches, such as marked sperm and sterile or sperm-less males [1, 2, 19].

In spite of what is known about the morphology and organization of mosquito spermatheca, particularly *Ae. aegypti*, it is currently not known if physiological differences exist between the large and the smaller spermathecae, or whether these spermathecae differ in terms of sperm allocation or sperm utilization [20, 21]. The characterization of molecules produced by the spermathecae and molecules directly associated with sperm viability can provide a further understanding of the function of these pivotal organs and may be used as targets for novel control approaches.

The present study was designed to provide a first look into the transcriptional profile of *Ae. aegypti* spermatheca, identifying unique or enriched transcripts associated with specific physiological roles. Our analyses were also focused on assessing transcriptional profiles both prior to (when the spermatheca is preparing to receive the male sperm) and after insemination (when the spermatheca allocates and preserves the sperm). Following RNA-seq analyses, eight differentially expressed mRNAs were selected, based both on their transcriptional profiles and putative roles, ranging from energy metabolism, to transcriptional regulation and hormonal signaling, to antimicrobial activity and ionic homeostasis. Additional criteria for inclusion of the eight transcripts in our downstream studies included: 1) differential expression levels between virgin and inseminated; 2) assigned predicted functional groups related to sperm maintenance; and 3) significantly higher expression (at least 30-fold higher) in the spermatheca compared to whole insect body. Selected transcripts were then used for “in situ*”* hybridization and RT-PCR to assess and confirm spatial and temporal expression profiles. Following RNAi-targeted knockdown (KD), our results indicate that disruption of expression of spermathecal transcripts associated with pre (virgin) and post (inseminated) mating events interfere with sperm viability and other physiological parameters linked to offspring production. This study points to the possibility of targeted approaches against molecules secreted in the spermatheca to reduce *Ae. aegypti* reproductive capacity.

## Results

### RNA-seq

Using RNA-seq, we generated a compendium of spermathecal transcripts (referred as “spermathecomes”) from virgin and from inseminated *Ae. aegypti* females. Paired-end sequencing was performed using the Illumina Hiseq 2000, resulting in over 21.1 million reads for virgin and over 19 million reads for inseminated females. After trimming (removal of low-quality residues < 20 bp), remaining reads for virgin and inseminated females were nearly 21 million and 19 million, respectively. Trimmed reads were mapped against the *Ae. aegypti* genome, resulting in 29.24 million coding sequences. Of all coding sequences, 22.5 million were localized to the spermathecae of virgin females representing 76.92% genome-wide coverage, and almost 22.7 million were localized to the spermathecae of the inseminated females representing 77.57% of the *Ae. aegypti* genome.

**Fig. 2:**
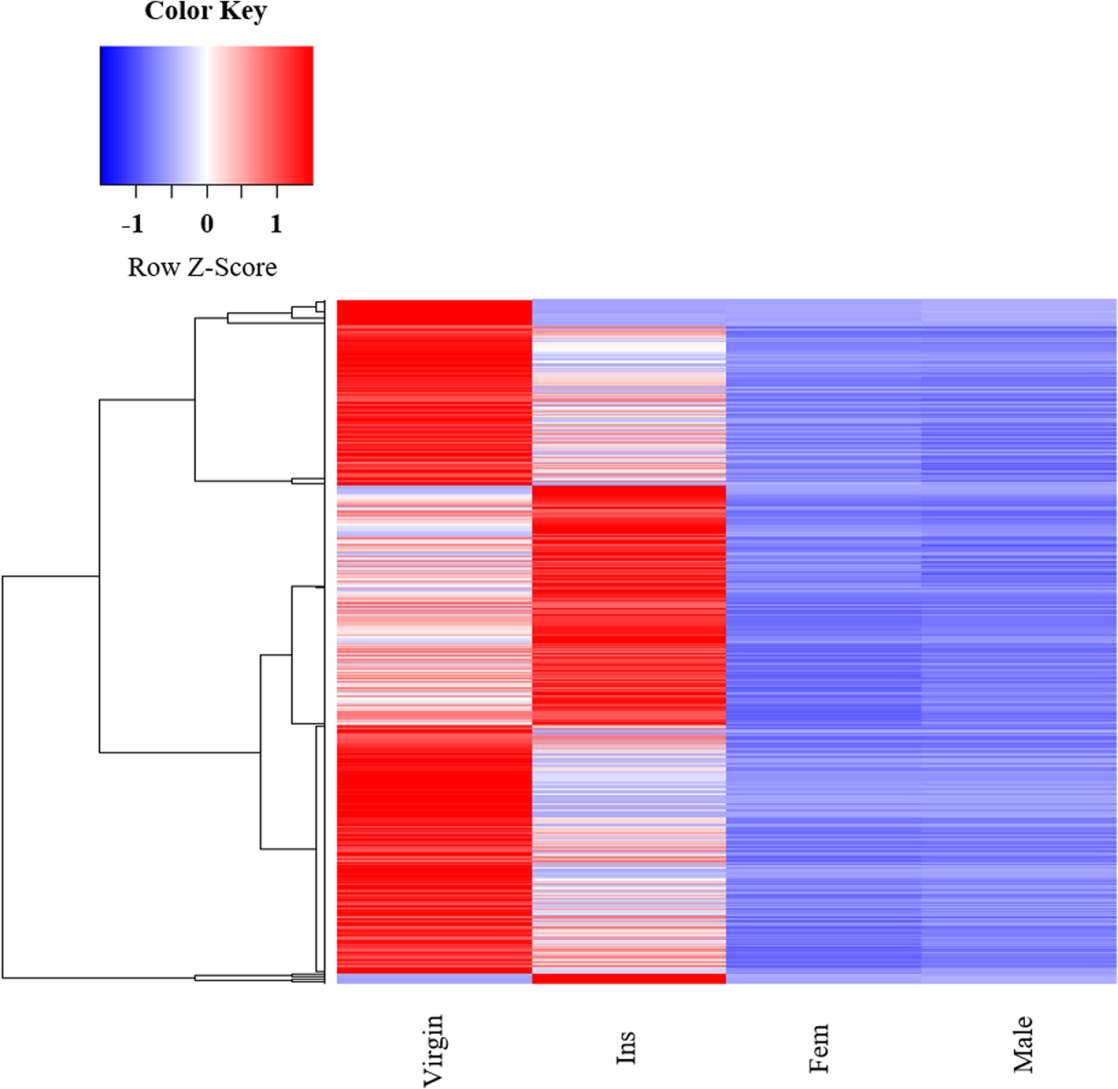
Upregulation of spermathecal genes in *Ae. aegypti*. The pattern of differentially expressed genes in female spermathecae from both virgin (Vir) and inseminated (Ins) females, and from male and female whole bodies. Z-score indicates transformed data from transcripts per million for each library. The lateral clusters represent the differentially expressed transcript groups, as shown in Additional file 1: Tables S1, S2, and S3

Expression levels of the spermathecal genes were separated from housekeeping genes by comparing the results from spermathecomes to whole body expression of female and male (F-test with *p*-value of 0.05 after Bonferroni correction for multiple comparisons). Transcripts were also analyzed using the RPKM normalization method for each mapped coding sequence. The index “maximum relative RPKM” was established as an indicator for the “expression index”. The total number of coding sequences was compared by their maximum relative RPKM (RPKM> 1), where RPKM = 1 corresponds to the value of the constitutive expression found in the whole body of both male and female, thus providing an enriched library for the two spermathecomes (virgin and inseminated). The transcripts identified in distinct clusters of the male and female differentially expressed genes (DEG) common to the spermathecomes, including genes overexpressed in the two spermathecomes, were grouped in a heat map graph representation (Fig. 2). The coding sequences were filtered and grouped according to their relative expression values among the samples (spermathecae versus whole female body), with at least double of the expression value (see Materials and Methods for Additional file 5).

To distinguish between the expression levels of the previously identified genes in the virgin or inseminated spermathecae, coding sequences were also compared among themselves and those whose expression differed by at least eight-fold were pre-selected (Additional file 1: Table S1). Of these pre-selected genes, 8044 (or 53%) transcripts were grouped into four functional groups: the unknown group (2744 genes or 18%), representing unknown gene functions, but conserved among the databases; the secreted group (2216 genes or 15%), with secretory signals or transcripts hypothetically released to the spermathecal lumen; and the signal transduction (1687 genes or 11%), and the metabolism (1398 genes or 9%) groups. A total of 661 DEG with at least an eight-fold increase over the expression levels of the housekeeping gene were identified, annotated, and divided into 21 functional classes (Additional file 1: Table S1). Of the 661 DEG identified, 111 were highly expressed (> 8-fold) in virgin spermathecae (Additional file 1: Table S1), with over 78% belonging to four functional groups/categories: extracellular matrix/cell adhesion (43 genes or 38%), secreted (27 genes or 24%), metabolism (9 genes or 8%), and signal transduction (8 genes or 7%) (Additional file 1: Table S3).

Unlike the previous comparison (virgin versus inseminated spermathecae), in the inverse comparison (inseminated versus virgin spermathecae), only 25 DEG were found with at least eight-fold increase. Of these, 70% were classified in four groups/categories: secreted (11 genes or 44%), unknown/conserved (3 genes or 12%), metabolism (3 genes or 12%), and signal transduction (2 genes or 8%) (Additional file 1: Table S4).

### Transcriptome validation and RT-PCR

From the RNA-seq results, we selected eight transcripts representing five functional groups/categories. Selection of transcripts was based on their expression levels (inseminated vs. virgin spermatheca) and predicted or assessed function in either the insect spermathecae or elsewhere in the reproductive system of female mosquitoes. Our selection also assumed a direct or indirect role of these transcripts in sperm maintenance in the spermathecae based on their functional categories and in light of their differential expression profiles assessed by the RNA-seq analyses. The following transcripts with their respective functional categories were selected for downstream analyses: *Ae-92,048* - glucose dehydrogenase or *Gld* (energy metabolism), *Ae-187,521* - chitin bind 4 or *ChtB4*, and *Ae-88,956*-chitin-binding domain type 2 or *ChtBD2* (chitin-associated), *Ae-27,176* - Atrophin-1 protein or *Atro-*1 (transcriptional regulation), *AeSigP-4002* - DHR4 ligand, *Drosophila Hormone Receptor 4 or DHR4* (hormonal signaling), *Ae-SigP-212,177* - N-acetylgalactosaminyl transferase 6 or *GALNT6* (enzymatic activity), *AeSigP-109,183* - Kazal-type serine protease inhibitor or *KSPI* (antimicrobial activity), and *AeSigP-66,427* - Na^+^/Ca^2+^ protein exchanger or *Na*^*+*^*/Ca*^*2+*^ (ionic homeostasis [22–24]) (Additional file 1: Table S5).

**Fig. 3:**
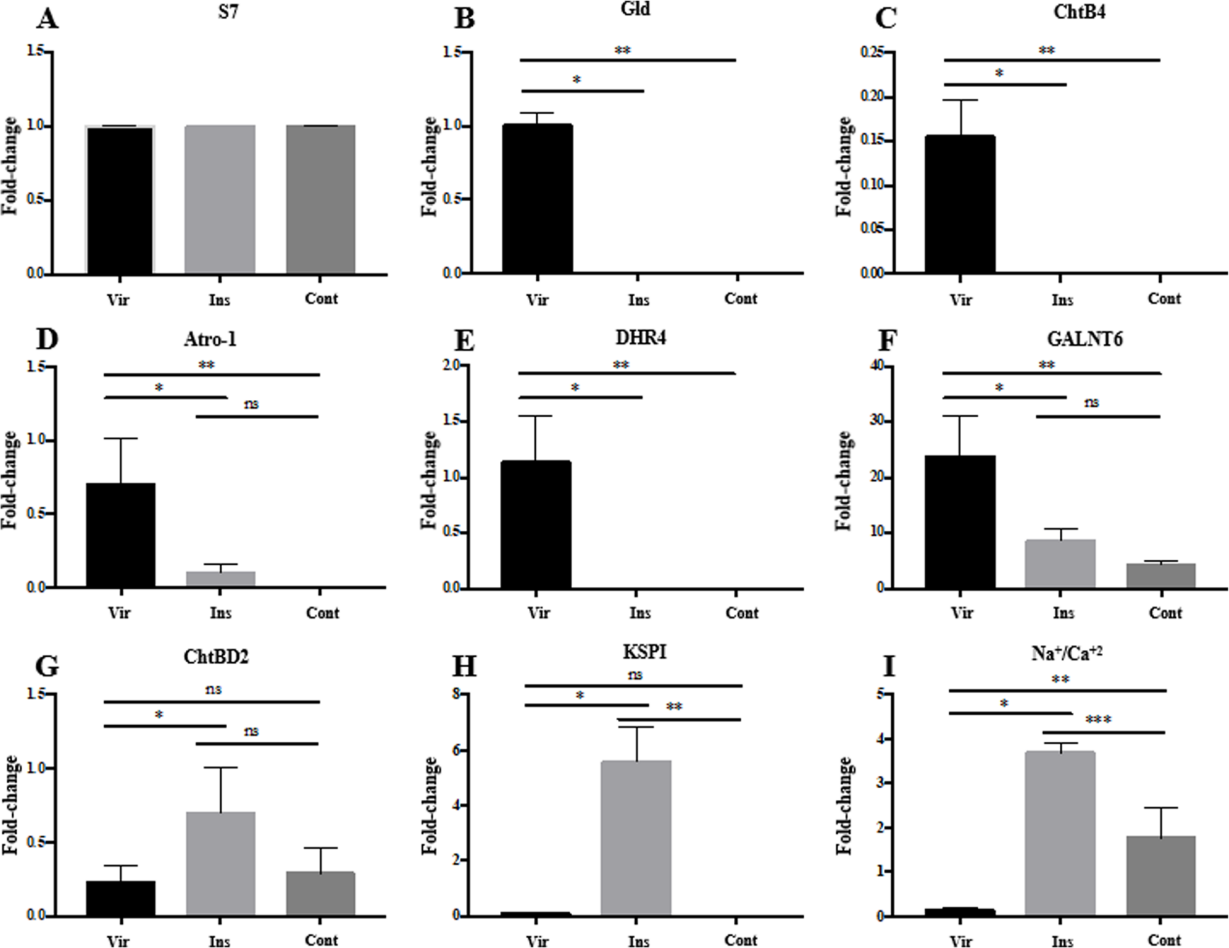
RT-PCR of genes expressed in *Ae. aegypti* spermathecae. Relative expression was determined in the spermathecae from virgin (Vir) or inseminated (Ins) females, and from the material collected in the spermathecal reservoir lumen (Cont) of inseminated females. Bar graphs show the fold-change of each sample normalized to S7 ribosomal gene. Reactions were done in triplicate using two biological replicates. Statistical analyses were performed using one-way ANOVA and Tukey’s multiple comparison test (α = 0.05). **a**: *S7* (*F* = 1; R^2^: 0.25; *P* = 0.4219), **b**: *Gld* (*F* = 477.2; R^2^: 0.9907; *P* < 0.001; *P < 0.001; **P < 0.01), **c**: *ChtB4* (*F* = 54.4; R^2^: 0.9236; *P* < 0.001; *P < 0.001; **P < 0.01), **d**: *Atro-1* (*F* = 17.24; R^2^: 0.793; *P* = 0.0008; *P = 0.0031; **P = 0.0011), **e**: *DHR4* (*F* = 29.27; R^2^: 0.8667; *P* = 0.0001. *P = 0.0003; **P = 0.0003), **f**: *GALNT6* (*F* = 21.91; R^2^: 0.8296; *P* = 0.0003. *P = 0.0021; **P = 0.0004), **g**: ChtBD2 (*F* = 5.724; R^2^: 0.5599; *P* = 0.0249; *P = 0.0303), **h**: *KSPI* (*F* = 75.8; R^2^: 0.944; *P* < 0.0001. *P < 0.0001; **P < 0.0001), **i**: *Na*^*+*^*/Ca*^*2+*^ (*F* = 74.28; R^2^: 0.9429; *P* < 0.0001. *P < 0.0001; **P = 0.0009; ***P = 0.0003)

The expression profile of each of the eight selected transcripts was assessed by RT-PCR in both the virgin and inseminated spermathecae, as well as the spermathecal content (i.e., the sperm within the reservoir lumen), and normalized to the expression levels of the *S7* gene (*AAEL009496-RA*) [25]. Spermathecal content was included to tease out gene expression in sperm present within the spermathecae. The fold-change expression values for all eight targeted transcripts varied depending on the physiological status (virgin vs. inseminated) and were consistent with the RNA-seq and the in silico analysis (Fig. 3).

Transcripts for *Gld* were downregulated after insemination, being undetected in the inseminated spermathecae and the reservoir content in comparison with the virgin spermathecae (*P* < 0.001). No difference was observed in *Gld* levels between the inseminated spermathecae and their respective reservoir content (*P* > 0.9999) (Fig. 3b). *ChtB4* was detected at low levels in virgin spermathecae only. No *Cht4* RNA transcripts were detected in either the inseminated spermathecae or the reservoir content (*P* < 0.01) (Fig. 3c). *Atro-1* was significantly downregulated in the inseminated compared with the virgin spermathecae (*P* = 0.0008), and was undetected in the reservoir content of the inseminated. No statistical difference was observed between the inseminated spermathecae and the reservoir content (*P* = 0.7164) (Fig. 3d). Expression of *DHR4* was, to some extent, similar to *Gld* in that *DHR4* levels were downregulated following insemination (*P* = 0.0001) (Fig. 3e). Expression levels of *GALNT6* were higher than all other transcripts. In the virgin spermathecae, *GALNT6* was significantly upregulated in comparison with the levels observed for both the inseminated spermathecae and their reservoir contents (*P* = 0.0003). No statistical difference was observed between the inseminated spermathecae and the reservoir content (*P* = 0.3933) (Fig. 3f). *ChtBD2* transcripts were identified in all the three samples (virgin, inseminated spermatheca, and reservoir content). However, for *ChiBD2*, comparing virgin and inseminated spermathecae, a higher expression was observed in the inseminated (*P* = 0.0249), and not significant when compared with the reservoir content (*P* = 0.0574) (Fig. 3g). For *KSPI,* there was a higher transcript expression in the inseminated spermathecae (*P* < 0.0001), and no difference between the virgin spermathecae and the spermathecal content (*P* = 0.9808) (Fig. 3h). The expression of *Na*^*+*^*/Ca*^*2+*^ was higher in the inseminated compared to virgin (*P* < 0.0001) and also higher in the reservoir content compared to the virgin spermathecae (*P* = 0.0009). Levels of *Na*^*+*^*/Ca*^*2+*^ were also higher in the inseminated spermathecae when compared to reservoir content (*P* = 0.0003) (Fig. 3i). (Additional file 1: Table S5) provides the summary, including the transcript code numbers, the related functional groups, primers used for RT-PCR, and the relative expression of each transcript for both the virgin and the inseminated spermathecae.

The expression profiles of the eight selected transcripts were assessed separately for midgut, ovaries, and carcasses (i.e., the body without gut, ovaries, and spermathecae) of both virgin (sugar-fed only, non-vitellogenic ovaries) and inseminated females (sugar and blood-fed, with developed/vitellogenic ovaries). In contrast to the results obtained for the spermathecae (Fig. 3), transcript abundance did not change between the carcasses of virgin and inseminated females (*P* = 0.5255). Additionally, no difference was detected regarding expression levels for the eight transcripts comparing ovaries before or after egg development of the inseminated females (and blood-fed). As expected, relative expression levels for the *S7* ribosomal protein transcript (*AAEL009496-RA*) remain unchanged between carcass, midgut, and developed and undeveloped ovaries (*P* = 0.5641) (Additional file 2: Figure S1A).

The expression levels for *Gld* (*P* = 0.1404), *ChtB4* (*P* = 0.3437), *DHR4* (*P* = 0.0922), *GALNT6* (*P* = 0.9336), *ChtBD2* (*P* = 0.5010), *KSPI* (*P* = 0.1875), and *Na*^*+*^*/Ca*^*2+*^ (*P* = 0.2298) were not significantly different between carcass, midgut, and undeveloped or developed ovaries (Additional file 2: Figure S1). In contrast, expression levels for *Atro-1* were significantly higher in developed ovaries (*P* = 0.0349) compared with carcass, midgut, and undeveloped ovaries (Additional file 2: Figure S1D).

### RNAi experiments

#### Knockdown effects on spermathecal-expressed genes

We used RNAi in an attempt to assess the role each selected gene play in the physiology of *Ae. aegypti*. Effects from dsRNA started on day one post-injection, with the peak in KD effect being observed 3 days after injection. As expected, relative expression levels for the *S7* ribosomal protein transcript remained unchanged among the 4 days following injection (*P* = 0.7567); however for the others analyzed genes, the inhibition peak in the gene expression levels was observed by the third post-injection day: *Gld* (*P* < 0.0001), *ChtB4* (*P* = 0.003), *Atro-1* (*P* < 0.0001), *DHR4* (*P* = 0.0009), *GALTN6* (*P* = 0.0019), *ChtBD2* (*P* = 0.0003), *KSPI* (*P* = 0.0496), *Na*^*+*^*/Ca*^*2+*^ (*P* = 0.0012) (Additional file 2: Figure S2).

dsRNA injections significantly reduced transcript levels for all eight targeted genes, with no significant differences between virgin and inseminated spermathecae after the injection (Additional file 1: Table S6). Fitness parameters, including overall survival, blood feeding, fertility, and egg morphology, as well as effects on the spermatheca morphology were assessed as a result of the dsRNA injections and are discussed separately below. A summary of the phenotypic effects provided by the KD effects for each target gene is shown in Table 1, and Additional file 1: Table S7.

**Table 1:** Summary of the phenotypic effects observed after dsRNA injection for each target gene of spermatheca of *Ae*. *aegypti*

Parameter	Genes highly expressed in virgin spermathecae (before mating)					Genes highly expressed in inseminated spermathecae (after mating)		
	*Gld*	*ChtB4*	*Atro-1*	*DHR4*	*GALNT6*	*ChtBD2*	*KSPI*	*Na*^*+*^*/Ca*^*+ 2*^
Female survival	–	↓	↓	–	–	–	–	–
Female oviposition	–	↓	–	–	↓	–	–	x
Fertility	–	↑	–	–	↑	↑	↑	x
Egg area	↑	↑	↓	↓	↓	↓	↓	x
Egg length	↑	↑	↑	↑	↑	↑	–	x
Fecundity	↑	–	–	–	↓	–	↓	x
Sperm motility within spermathecal reservoir	–	–	–	–	–	–	–	↓

Up (↑) and down (↓) arrows represent an increase or a decrease for each respective parameter indicated. “x” indicates complete abrogation of egg development, in which the parameters could not be analyzed. “–” indicates no difference

### Survival analysis

Female mosquito survival was compared between females injected with dsRNA-targeting genes putatively associated with spermathecal function and females injected with control dsRNA (dsEGFP). The survival assays considered virgin and inseminated females (based on the higher expression of the selected genes for each case) to assess KD effects during the lifetime of the female (Additional file 2: Figure S9). For this, dsEGFP control was injected on days one and two after emergence. Mosquito survival was assessed for 10 days subsequent to dsRNA injections (Additional file 2: Figure S3).

When compared with the dsEGFP-injected control, no difference between the survival was found for dsGld (*P* = 0.6201), dsDHR4 (*P* = 0.6986), dsGALNT6 (*P* = 0.2378), dsChtBD2 (*P* = 0.3739), dsKSPI (*P* = 0.2996), and dsNa^+^/Ca^2+^ (*P* = 0.3106). However, the survival was reduced in the dsRNA treatments for *ChtB4* and *Atro-1* compared to control (*P* = 0.0364 and 0.0109, respectively).

### Fertility analysis

We assessed the effect of dsRNA injections on female oviposition and fertility after blood feeding to determine whether KD affected the spermathecae/sperm only, or whether non-spermathecal tissues of the reproductive system were also affected. Although the proportion of females that laid eggs was not affected in dsGld, dsAtro-1, or dsDHR4 experimental groups (*P* = 0.9024, *P* = 0.9024, *P* = 0.4343, respectively), the number of females that laid eggs was negatively affected following injections with dsChtB4 (*Ae-187,521*) or with dsGALNT6 (*P* = 0.00489 and 0.0179, respectively) (Additional file 2: Figures S4A and S4B). dsRNA targeting *Na*^*+*^*/Ca*^*2+*^ inhibited egg laying completely (Table 1). Curiously, however, among the females that effectively laid eggs following the dsRNA injections and blood feeding, those injected with dsChtB4, dsGALNT6, dsChtBD2, and dsKSPI laid more eggs than the control group (dsEGFP) (*P* = 0.0489, 0.0179, 0.0235, 0.0455, respectively) (Additional file 2: Figure S4C and S4D).

### Egg morphometry

When counting the mosquito eggs to assess KD effects on fecundity, we noticed a difference in egg morphology. We then measured both length and total area of eggs laid to determine if such changes could be associated with embryo survival compared with dsEGFP-injected control. Females injected with dsRNA targeting *Gld*, *ChtB4*, *Atro-1*, *DHR4*, *GALNT6,* and *ChtBD2* laid eggs that were longer (*P* < 0.0001) (Additional file 2: Figures S5A and S5B), but no differences in either length or area were observed in the eggs laid by females injected with dsKSPI (*P* = 0.9550 and *P* = 0.9991, respectively) (Additional file 2: Figure S5B). For dsGld-injected females, the area of the eggs laid was larger than the area of eggs laid by the control females (*P* < 0.0001), whereas for all the other treatments the area of the eggs laid was smaller than the control-laid eggs (*P* < 0.0001) (Additional file 2: Figures S5C and S5D).

### Fecundity

Mosquito fecundity was measured considering the number and the viability (hatching) of the eggs laid by the dsRNA-injected females (Additional file 2: Figure S6). Injection with dsRNA targeting *Gld*, *GALNT6,* and *KSPI* decreased egg hatching (*P* = 0.0365, *P* = 0.0002, and *P* = 0.0008, respectively). Unlike the other injections, dsRNA targeting *Na*^*+*^*/Ca*^*2+*^ affected egg development of *Ae. aegypti* females as their ovaries did not develop even up to 7 days after the blood feeding (Additional file 2: Figure S7 and Additional file 6: Movie S1). Moreover, 5 days after blood feeding, the *dsNa+/Ca2 +* −injected females laid no eggs*.* Notwithstanding, the presence of fecal stains on the filter paper or substrate used for egg laying, checked for both virgin and inseminated females, was indicative of complete blood digestion (Additional file 2: Figure S8).

### Morphology of spermatheca and stored sperm

To identify any effects of the dsRNA injections on the spermathecal morphology and sperm integrity, overall spermathecae and sperm morphologies (for sperm inside the spermathecae) were investigated. The morphologies of the spermathecal duct, the glandular portion, and the reservoir were not altered by the injections. Surrounding the internal part of the reservoir and continuously with the spermathecal duct, a well-structured thicker cuticle was observed (Additional file 3).

Under normal conditions following insemination, spermatozoids are organized circularly within the reservoir lumen, arranged parallel to each other [10,11], and with typical motility (Additional file 3 and Additional files 5, 6, 7, 8 and 9: Movies). In contrast, we observed reduced sperm motility 1 day after mating in females injected with dsNa^+^/Ca^2+^ (Additional files 7 and 8 Movies S2 and S3). Curiously, that was followed by no motility for spermatozoids within the inseminated spermathecae, 5 days following blood feeding (Additional file 9: Movie S4). However, as the reservoir was mechanically broken with forceps, the released sperm appeared to swim normally (Additional file 10: Movie S5). A summary of the measurements of the dsRNA-injected females and the controls are presented in Additional file 1: Table S7.

### RNA in situ hybridization

**Fig. 4:**
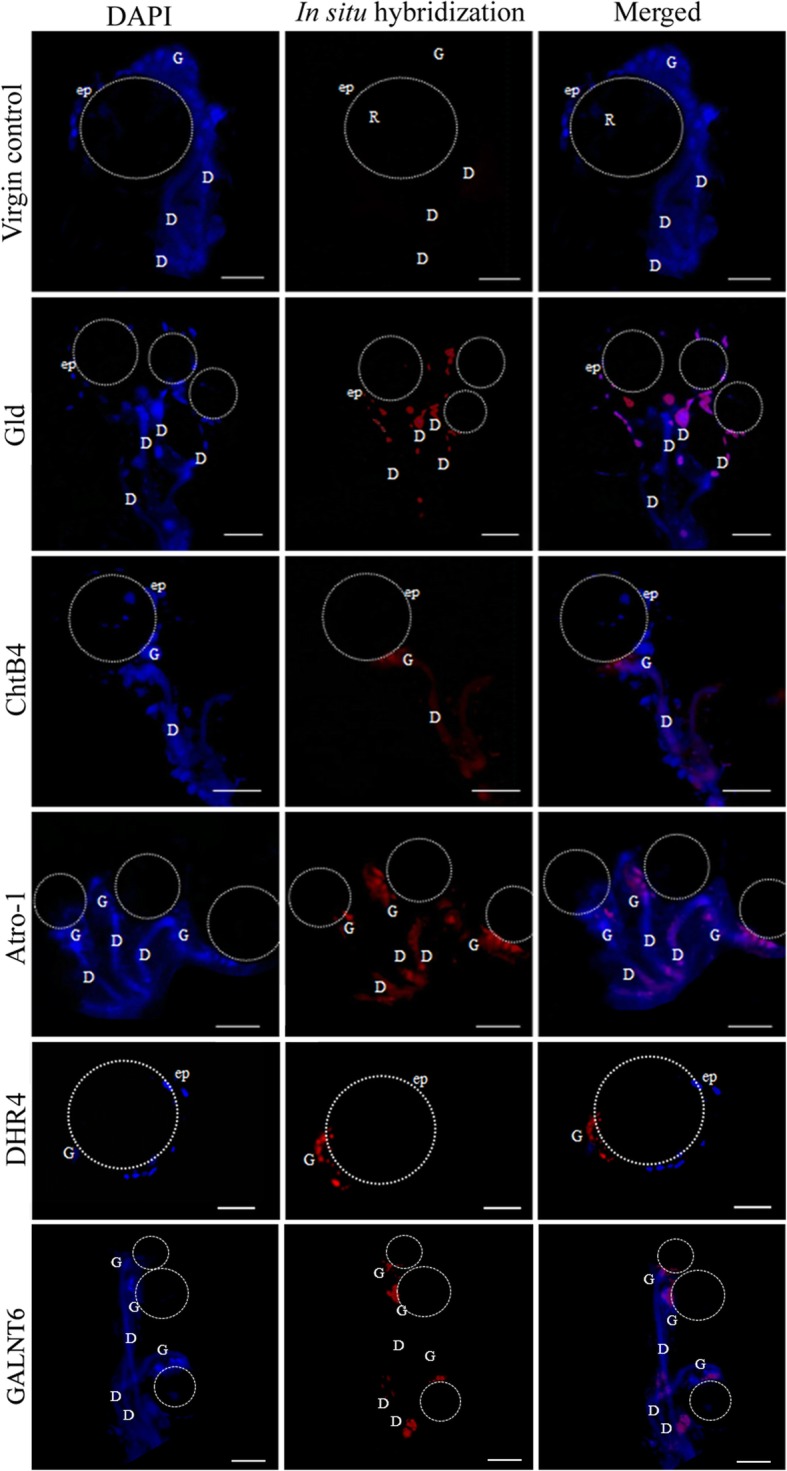
Detection of gene transcripts *Gld*, *ChtB4*, *Atro-1*, *DHR4*, and *GALNT6* in whole mounts of spermathecae of *Ae. aegypti* (virgin females) by in situ hybridization with red RNA probes and DAPI (blue). EGFP probe was used as control. (D) spermathecal duct, (G) spermathecal gland, (dc) spermathecal duct cells, (ep): epithelial cells, dotted line: spermathecal reservoir. Bar: 50 μm

**Fig. 5:**
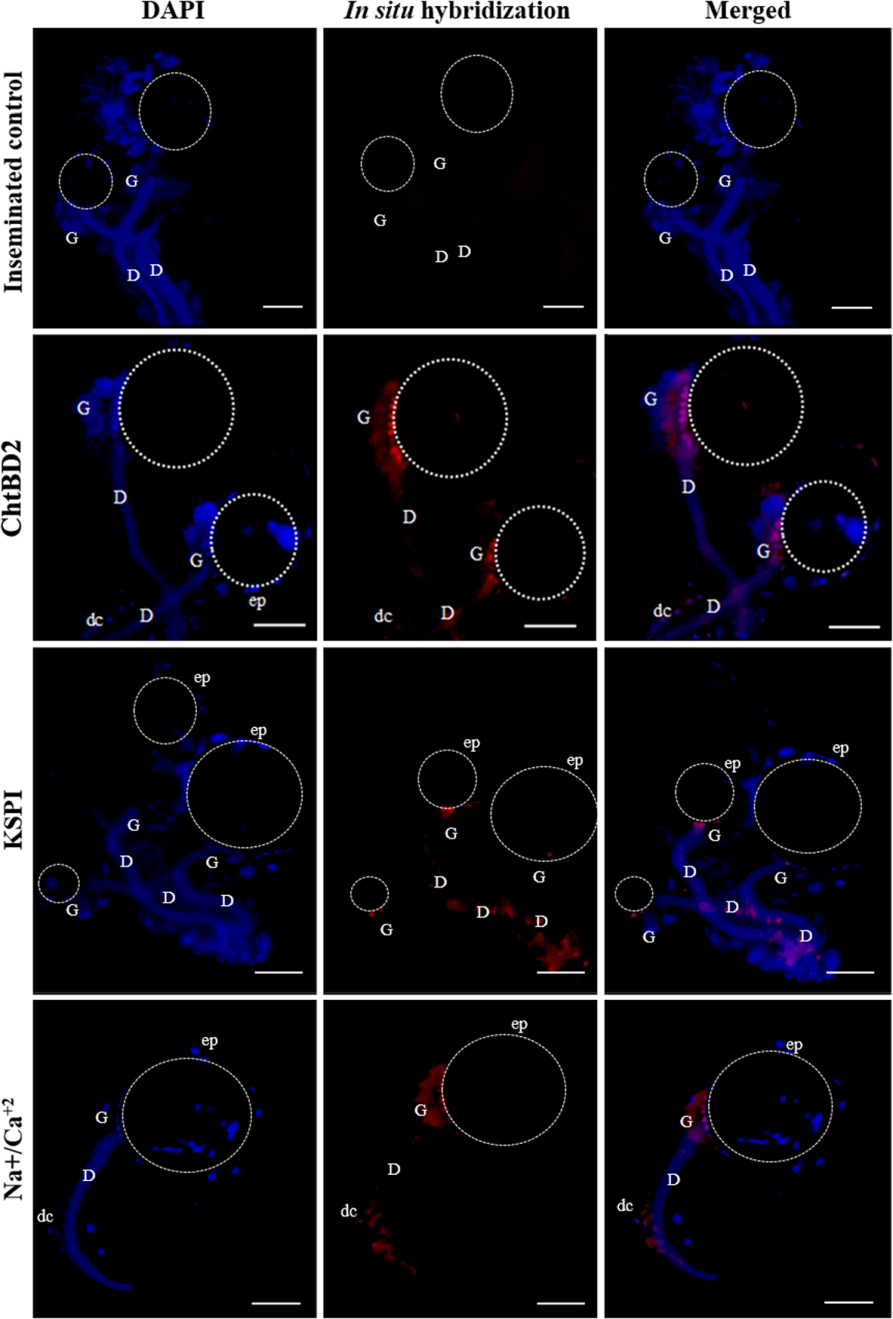
Detection of gene transcripts *ChtBD2*, *KSPI*, and *Na*^*+*^*/Ca*^*2+*^ in whole mounts of spermathecae of *Ae. aegypti* (inseminated females) by in situ hybridization with red RNA probes and DAPI (blue). EGFP probe was used as control. (D): spermathecal duct, (G): spermathecal gland, (dc): spermathecal duct cells, (ep): epithelial cells, dotted area: spermathecal reservoir. The spermathecal cuticle did not allowed the visualization of stained sperm. Bar: 50 μm

Next, we used in situ hybridization of whole spermathecae mounts labeled with specific RNA sequences to ascertain the location(s) within the spermathecae where the eight selected target genes were being expressed. For *Gld*, the fluorescence signal was detected along the spermathecal duct, with a higher intensity in the duct of individual glandular cells. Additionally, the fluorescent signal was detected in some epithelial cells of the reservoir (Fig. 4). For *ChtB4*, the fluorescence signal was detected in the spermathecal duct and at the site of attachment of the glandular cells to the duct. The fluorescence intensity of the probes was higher at the attachment site of the duct of the spermathecal reservoir, where the spermathecal gland is located (Fig. 4). *Atro-1* was detected in the gland, mainly close to the reservoir cuticle, and in the duct (Fig. 4). *DHR4* was detected only in the glandular cells, in the apical portion associated with the ductule (Fig. 4). *GALNT6* was detected in the spermathecal gland and with low intensity in the spermathecal duct (Fig. 4). For *ChtBD2,* the transcripts were detected in the spermathecal glandular portion, next to the reservoir cuticle (Fig. 5). The *KSPI* transcripts were detected in the spermathecal duct and at the site of attachment of the glandular cells to this duct. *Na*^*+*^*/Ca*^*2+*^ were mostly detected in the spermathecal glandular portion close to the reservoir cuticle and the spermathecal duct, close to the oviduct opening (Fig. 5). A summary of the fluorescence signals provided by the in situ hybridization for each target gene is shown in Table 2.

**Table 2:** Fluorescence intensity after in situ hybridization in whole mounts of spermatheca of *Ae*. *aegypti*

Gene ID number	Putative function	Spermathecal duct epithelium	Glandular cells of spermathecal duct	Spermathecal gland	Reservoir epithelium
*Ae-92,048*	Glucose dehydrogenase (Gld)	**+**	**–**	**+++**	**++**
*Ae-187,521*	Chitin bind 4 (ChtB4)	**+**	**+**	**++**	**–**
*Ae-27,176*	Atrophin-1 protein (Atro-1)	**+**	**++**	**+++**	**–**
*AeSigP-4002*	DHR4 ligand (DHR4)	**–**	**–**	**++**	**–**
*AeSigP-212,177*	N-acetylgalactosaminyl transferase 6 (GALNT6)	**–**	**+**	**+++**	**–**
*Ae-88,956*	Chitin-binding domain type 2 (ChtBD2)	**–**	**–**	**+++**	**–**
*AeSigP-109,183*	Kazal type serine protease inhibitor (KSPI)	**++**	**++**	**++**	**–**
*AeSigP-66,427*	Na^+^/Ca^2+^ exchanger protein (Na^+^/Ca^2+^)	**+++**	**+**	**+++**	**–**

The assays were performed for each transcript, and staining measurements were taken in different portions of the spermathecae. Different levels of staining intensity (+) or lack thereof (−) are indicated

## Discussion

Our in silico analyses identified genes differentially expressed in spermathecae from virgin and inseminated females, leading to the identification of functional groups associated with energy metabolism, cell adhesion, gene expression machinery, and detoxification [26]. Notably, a greater number of highly expressed genes were identified in the virgin spermathecome (111 DEG) than in the inseminated spermathecome (25 DEG). Such upregulation in gene expression in virgin spermathecae likely prepares the organ for the arrival and its ability to maintain the male sperm. As female mosquitoes mate only once, successful maintenance of the sperm must continue during the female lifespan. Similar events were previously shown for *An. gambiae* [27] and also for *Crematogaster osakensis* queen ants [18], and associated with sperm maintenance and viability.

Our gene knockdown analyses revealed insights specific to each of the eight selected transcripts. Although not all transcripts led to significant loss of the various fitness parameters investigated, their effects were uniquely representative of their role in various aspects of the physiology of the spermathecae.

*Gld* is an important carbohydrate-metabolizing enzyme related to the glucose-trehalose conversion pathway. Disruption of the trehalose metabolism, such as from a lack of *Gld*, can severely affect sugar metabolism [13, 28, 29]. Though we did observe an increase in the length and the area of the eggs following *Gld* KD, fecundity and fertility remained unaffected. In spite of the role *Gld* plays in insect trehalose metabolism [28, 29], and possibly in insect reproduction [13], KD of *Gld* led to no marked effects in *Ae. aegypti* reproduction.

Two of the transcripts selected are associated with the formation of a chitin layer. Besides being a major component of the insect cuticle, chitin also lines the spermathecal reservoir, forming a continuous cuticular layer with the spermathecal duct. The precise role (or multiple roles) played by such chitin layer in gamete maintenance is not fully understood [6]. However, following *ChtB4*-KD, a reduction in mosquito survival was observed and possibly associated with changes in the protective cuticular layer [30–32].

The RNA in situ hybridization signal for both *ChtB4* and *ChtBD2* indicated that these transcripts are present near the glandular ductules, which may be related either to the glandular cells, or the epithelial cells, or both. These two types of cells are physically associated with *Ae. aegypti* spermathecae [10, 11]. The glandular ductule of the glandular cells of the spermatheca is internally covered by a thin layer of chitin continuous with the reservoir cuticle, supporting the hypothesis that the proteins that have an affinity for chitin are expected to be secreted into the extracellular space [32].

*Atro-1* is an atrophin family co-repressor required for embryo development [33]. *Atro-1* negatively regulates the epithelial growth factor receptor (EGFR) that promotes the development of imaginal discs, precursor of ectodermic tissues of insects [34–36]. The high levels of expression of *Atro-1* in virgin spermathecae suggest that its expression precedes sperm storage in *Ae. aegypti*. Because the spermatheca is an ectodermic organ, we investigated if the KD of *Atro-1* interferes with its functioning at the beginning of female reproductive life, the period in which sperm storage occurs. Atro-1 is likely involved in vital pathways, as KD of *Atro-1* led to lower overall survival in *Ae. aegypti* (this study) and also in *Blattella germanica* [37]. However, it does not appear that Atro-1 is directly involved in fecundity or fertility of females as no significant differences were observed between *Atro-1* KD and control.

High levels of nuclear receptors (NR) transcripts were present in virgin spermathecae. Our finding that *DHR4* transcripts were only detected in virgin females raised the possibility that this receptor is related to changes in the physiology after *Ae. aegypti* insemination. *DHR4* expression in *Ae. aegypti* spermathecae was detected in the glandular portion, near the reservoir cuticle, consistent with that described in *D. melanogaster* during metamorphosis when *DHR4* is found primarily in cell nuclei [38]. Considering the abundance of *DHR4* transcripts in the virgin spermathecae, we investigated whether the KD of *DHR4* in *Ae. aegypti* would interfere with gametes storage and female fitness. *DHR4* regulates development (e.g., molting) in *D. melanogaster* mediated by the steroid hormone ecdysone [39]. In *An. gambiae*, it has been shown that male semen transferred to the female genital tract contains the steroid hormone 20E, which induces changes in sexual behavior in *[*40*]. Hence, DHR4 may be a potential target against 20E-triggered signaling or regulation in mosquitoes. Following mating and insemination, DHR4 levels were significantly reduced* suggestive of a role related to changes in female behavior after mating, including reduced receptivity to males.

The *GALNT6* enzyme is part of the UDP-N-acetylglucosamine (UDP-GlcNAc) pathway, which is involved in the maintenance of the exoskeleton of insects [41]. In *Tribolium castaneum*, *GALNT6* expression was reported in other adult tissues such as the midgut, fat bodies, ovaries, and testis [30]. An abundance of *GALNT6* was detected in the spermathecae of virgin *Ae. aegypti,* but absent from the spermathecae of inseminated females. *GALNT6* transcripts were detected in the spermathecal gland and spermathecal duct glandular cells but with low signal intensity. Chitin (and chitin metabolism) is possibly essential for maintenance of secretory ductules of the glandular cells that release the glandular secretion into the spermathecal lumen [32]. Based on the different expression profiles, we reasoned that *GALNT6*-KD may lead to disruption of the chitin metabolism affecting the spermathecal organization in virgin females.

Following *GALNT6*-KD, we observed a reduction in the number of females that laid eggs. However, the number of eggs laid was actually higher than the control. Thus, in spite of the decrease in egg production by some females, the *GALNT6*-KD had neither an effect in offspring production nor it led to changes in spermathecal morphology or its capacity to store sperm. Our results aligned at least partially with those described for *Rhodnius prolixus* (Heteroptera) following knockdown of *chitin synthase*, causing a 60% reduction in oviposition and altered egg morphology [42].

*AeSigP-109,183*, a putative Kazal-type serine protease inhibitor (*KSPI*), was the most abundant transcript identified in the inseminated spermathecae. *KSPI* is a member of a family of proteins involved in preventing or regulating proteolysis [43, 44]. Another *KSPI*, AaTI, previously identified in the salivary glands and the midgut of *A. aegypti* is thought to have a role in mosquito innate immune response [45]. The high levels of the expression of *KSPI* (*AeSigP-*109,183) in the inseminated spermathecae may either be associated with maintaining homeostasis by inhibiting unregulated proteolysis in the spermatheca reservoir that may lead to sperm damage [46] or protecting it from potential pathogens transmitted during mating (i.e., venereal transmission).

Surprisingly, *KSPI*-silenced females laid significantly more eggs than the controls. However, the eggs were smaller and with a clear loss of viability. *KSPI* expression was predominantly found along the spermathecal duct close to the common opening of oviduct. Taking into account that Kazal serine proteases are involved in antimicrobial activity (by inhibiting protease activity of pathogens) [44, 45], the expression of *KSPI* is potentially associated with protection of the sperm from pathogens during their journey within the duct.

Lastly, we investigated the Na^+^/Ca^2*+*^ exchanger *AeSigP-66,427*. This transcript is highly expressed in the inseminated spermathecae and is also present in the content of the reservoir (i.e., in sperm). However, *AeSigP-66,427* is not present in the virgin spermathecae. Such high level of *AeSigP-66,427* in the inseminated spermathecae compared with the reservoir content may also be the result of an additive effect of the sperm inside the reservoir and spermathecal tissues. *AeSigP-66,427* KD led to clearly detectable changes in fertility. The motility of the sperm inside the reservoirs was also severely affected following KD, and this effect lasted for at least 7 days (i.e., last data collection point beyond which the RNAi effect likely weans). The motility resumed when the reservoir shell wqas broken releasing the sperm, suggesting that the effect on motility is temporary or that the sperm motility is not completely impaired by the KD. In combination with the morphological assessment, such results indicate that the spermatozoids present in the *AeSigP-66,427* KD females were alive but unable to swim within the small space of reservoir lumen [100 μm or 75 μm diameter [10, 11]] and their movement from the lumen to the common oviduct was also impaired, thus affecting fertilization.

Ion imbalance in the spermathecae has been previously linked to the presence of non-motile sperm in *A. mellifera* [47, 48]. The lack of the Na^+^/Ca^2*+*^ exchange caused by the KD of *AeSigP-66,427* thus support the idea of an ionic basis for control of sperm motility and longevity [22–24, 47–49]. The impact on the ion exchange homeostasis regarding the spermathecal microenvironment was likely due to the KD of *AeSigP-66,427* in glandular and epithelial cells, as well as the gametes, and is supported by our findings of the in situ hybridization (positive signal) and by the presence of the transcript in the spermathecal content.

Oocytes accumulate yolk protein precursors whose uptake is directly regulated by Na^+^/K^+^ ATPases in *Locusta migratoria* [50]. Additionally, Ca^2+^ ionic channels play an important role in vitellogenin (Vg) uptake in insects, including *Ae. aegypti* [51, 52]. Any alteration in this ionic balance is likely to result in egg Vg storage reduction and impairment of egg development. In the *AeSigP-66,427 Ae. aegypti* KD females, there was no evidence of egg development even after blood feeding. We hypothesize that lack in egg development in these females was due to a disruption, at least temporarily, in the acquisition of nutrients from the blood meal [53].

As major vectors of important human pathogens, mosquitoes impose an enormous burden on public health. *Aedes aegypti* is a primary vector for many human diseases. The successful ability of this mosquito as disease vector is directly associated with its high reproductive output. Mosquitoes mate only once, and a single mating event provides enough sperm to fertilize the eggs for the entire reproductive life of the female [20]. The spermathecae play a crucial role in this process, providing a suitable environment, physical protection, and nutrient supply (reviewed by 6). Hence, a better understanding of the mechanisms that promote successful storage of sperm in the spermathecae may unravel potential targets for the reduction of vector populations in the field and lowering the burden of mosquito-borne diseases such as dengue and malaria.

## Conclusions

This study provides a unique catalog of spermathecal transcripts from virgin and inseminated *Ae. aegypti,* and highlights aspects of the critical balance between spermatheca gene expression regulation, male sperm viability, and overall insect fertility. In spite of the non-linear and target-dependent effects from our KD experiments, our results provide evidence of the role played at least for some of the selected transcripts in female survival, egg production, and fecundity. Knockdown of the Na^+^/Ca^2^ exchanger *AeSigP-66,427* in particularly provided strong evidence for the role of this transcript in sperm motility and fecundity. Because the mosquito spermatheca directly influences sperm viability and thus female fertility, understanding of underlying mechanisms related to sperm maintenance and survival by the spermatheca will likely identify potential targets for intervention and vector population control.

## Methods

### Ethics statement

This study was performed in accordance with the recommendations in the Guide for the Care and Use of Laboratory Animals of the National Institutes of Health. The animal use was approved by the Johns Hopkins University Institutional Animal Care & Use Committee (IACUC) (Protocol M018H18), the Johns Hopkins Institutional Biosafety Committee (IBC) (Protocol #DN0305070116), and the Ethics Committee of Universidae Federal de Viçosa (UFV-Protocol 561/2016).

### Sample preparation and RNA-seq

*Aedes aegypti* females (strain PPCampos, Campos dos Goytacases, Latitude: − 21.7545, Longitude: − 41.3244 21° 45′ 16″ South, 41° 19′ 28″ West, Rio de Janeiro, Brazil) were obtained from a colony maintained in the Departamento de Biologia Geral, Universidade Federal de Viçosa (DBG/UFV), Brazil. For our studies, stored eggs were allowed to hatch in dechlorinated tap water under a 12 h photoperiod at 25 °C ± 0.5 °C. Mosquito larvae were fed with turtle food (Reptolife®), and upon emergence, adults were fed on 10% sucrose solution ad libitum*.* Adult females were separated on the day of emergence into two cages/groups, one cage containing only females (virgin females) and another cage containing both males and females (inseminated females), at a two-to-one ratio of males to females. Seven days after emergence, the spermathecae (including all parts: reservoir and its content, spermathecal duct, and glandular cells – Fig. 1) from 600 females of each group were dissected in RNAse-free PBS solution (0.1 M, pH = 7.6), collected into microcentrifuge tubes containing 1 mL of TRIzol® (Invitrogen, Carlsbad, CA), homogenized, and stored at − 70 °C until the RNA extraction. The number of spermathecae collected from each group of females was 1800. All instruments used during dissections, including needles, stereomicroscopes, forceps, and slides, were wiped with RNAse AWAY® (Sigma-Aldrich, Buchs, Switzerland). Total RNA was extracted from each of the two spermathecae pools (1800 per pool) according to the manufacturer’s protocol. The RNA quality was confirmed in 1% agarose gel with 1% XT MOPS (Bio-Rad, Hercules, CA) and 5% formaldehyde. The RNA integrity was also confirmed using an Agilent 2100 Bioanalyser® and the RNA 6000 Nano chip (Agilent, Waldbronn, Baden-Württemberg, Germany). RNA quantification was performed using Qubit 2.0 Fluorometer® (Thermo Fisher Scientific). The degree of purity (260 nm/230 nm/280 nm) was confirmed using a NanoDrop ND-1000 (Thermo Fisher Scientific).

RNA libraries were prepared with 400 ng of high-quality total RNA using the TruSeq RNA Sample Preparation v.2 kit (Illumina®) diluted to 10 nM/μL (according to manufacturer instructions) and divided into two libraries (technical replicates) per pool. Each pool was sequenced using the MiSeq reagent v3 kit (600 cycles/paired-ends) in MiSeq Illumina (http://www.illumina.com/products/truseq_rna_library_prep_kit). The read length average was 300 bp. RNA library preparation and sequencing were performed at the Kansas State University’s Integrated Genomic Facility.

### RNA-seq analysis

Transcriptome analysis of whole spermathecae was performed according to Ribeiro et al. [54, 55]. Briefly, the Fastq data provided by the sequencing were quality- and primer-trimmed, excluding reads smaller than 20 bp. Read files were concatenated and assembled in a single-ended mode using Abyss [56] and Soapdenovo-Trans [57], with a k-parameter set between 21 and 91 in increments of 5 [54] with a 3′ prime poly-A enrichment. Fasta-generated files were added to the Vector-Base’s *Ae. aegypti* coding sequences (version 3.3) and assembled using PSI- BLAST and CAP3 pipeline [58]. Coding sequences were extracted based on the presence of signal peptide, open reading frame (ORF), and by similarities with related proteins available at RefSeq (invertebrate), an NCBI database, Diptera proteins deposited in GenBank (NCBI), and SwissProt. To check the transcript levels related to the physiological condition (i.e., virgin and inseminated spermathecae), the whole body’s housekeeping gene expression were excluded in the assembled transcriptomes; the expression values of the transcripts from the whole body of virgin sugar-fed males and females were deposited in the Sequence Read Archives (SRA) of the NCBI BioProject PRJNA261799 (Liverpool strain) [59]. Each spermathecal library was mapped to identified coding sequences, available on the databases, using BLASTN with a word size of 25, one gap, and allowing the identity of 95% or higher. Up to five matches were permitted if and only if the scores were the same as the most substantial score. A chi-squared test was applied for each coding sequence to detect statistical differences between the paired-reads. The Bonferroni and FDR correction [60] were applied using the *P*-value package version 3.3.0 from R software [61]. The normalized reads rate was determined by the expressions r1 x R2 / [R1 x (r2 + 1)] e r2 x R1/ [R2 x (r1 + 1)]; where, r1 and r2 are the reads for each library (virgin and inseminated spermathecae) mapping to a particular transcript, and R1 and R2 are the number of total reads from the libraries mapped over all identified coding sequences. One unit was added to avoid division by zero. An “expression index,” defined as the number of reads mapped to a particular coding sequence multiplied by 100 and divided by the highest number of mapped reads to a particular coding sequence, was established [62]. The RPKM and TPM values were calculated for each mapped library [63]. To compare the gene expression over the libraries, we applied the TPM index, and for absolute expression values we used RPKM values or the normalized read index, as described above. Heat map graph was done with the program heatmap2, from the gplots package within the R software package, with default parameters and using Z scores for data normalization [64]. Protein annotation was automated and done based on a vocabulary of approximately 290 words found associated with several databases, including NCBI NR light, SwissProt, Gene Ontology, CDD, KEGG, KOG, Pfam, SMART, RefSeq-invertebrates, REPBASE-RPS, rRNA, and a subset of GenBank sequences containing Diptera (organism). The absence of a signal peptide and transmembrane domains were also considered during annotation. Detailed bioinformatics analyzes can be found in Karim et al. [58].

### Transcriptome validation and RT-PCR

Validation of RNA-seq was performed using RT-PCR to assess the expression profiles of the eight selected transcripts (Additional file 1: Table S5), following the bioinformatics analysis of the spermathecal transcriptome. Here, the virgin and the inseminated spermathecae were obtained, as before, from *Ae. aegypti* (*Rockefeller* strain) available in the Department of Immunology and Microbiology (DIM) at the Johns Hopkins University (Baltimore, MD). Following hatching in distilled water, larvae were fed with Cat Chow (Purina®) and maintained under the conditions indicated above. Another group of females was again separated into two groups (virgin and inseminated) and dissected 7 days after emergence. One hundred virgin and inseminated females (300 spermathecae each) were dissected in RNAse-free 0.1 M PBS (pH 7.6), and the total RNA was extracted using TRIzol® (Invitrogen, Carlsbad, CA). To tease out gene expression in sperm present in the spermathecae, expression levels of the same selected transcripts were also assessed in the luminal content of the spermathecae from inseminated females. For this, reservoirs of 100 inseminated females were disrupted by hand (using forceps), transferred to 1.5 mL microtubes, containing 100 μL of PBS solution, and centrifuged at low speed (below 3000 rpm/956 *g* for 10 s) to avoid cell damage. The supernatant with the reservoir contents was subjected to total RNA extraction, cDNA synthesis, and expression quantification together with the virgin and the inseminated spermathecae.

To confirm spermatheca-specific profile, the expression profile of each of the eight selected transcripts was also assessed in carcasses (i.e., the body without guts and ovaries), midguts, and ovaries from virgin (non-blood fed) and inseminated (blood fed) females. Carcasses, midguts, and ovaries were collected from 5-day old virgin or inseminated females. Developed ovaries were collected 2 days after the blood meal following feeding on an anesthetized mouse, and as described in the RNAi experiments section below. Total RNA was extracted separately for each sample belonging to the two pools (virgin vs. inseminated), and each pool contained tissues from ten females. Each RNA sample was treated with DNAse I (Invitrogen), precipitated in ethanol/ammonium acetate solution, and finally suspended in RNAse-free water. After RNA quantification (using NanoDrop Lite Spectrophotometer, Thermo Fisher Scientific), 1st strand cDNA was obtained for each sample using Superscript III (Invitrogen) with random hexamers (Thermo Fisher Scientific) and 500 ng of RNA per sample. cDNAs were treated with RNA H (New England Biolabs) for 10 min at 37 °C, and stored at − 70 °C until use.

Relative gene expression profiles were assessed in real time, using SYBR Green PCR Master Mix (Applied Biosystems, Thermo Fisher Scientific) in 20 μL reactions containing 300 nM of each primer, and 100 ng each cDNA. PCR were run using MicroAmp® Fast Optical 96-Well Reaction Plate with Barcode (0.1 mL) (Applied Biosystems, Life Technologies) in the StepOne™ Real-Time PCR System (Applied Biosystems, Life Technologies). The amplification conditions were 94 °C for 2 min, 94 °C for 15 s, 60 °C for 1 min, 95 °C for 15 s, 60 °C for 1 min, and 95 °C for 15 s. Each reaction was performed in triplicate using two biological replicates.

The relative expression profiles were determined using Real Time Quantitative PCR and the 2^-ΔΔCt^ Method [65]. The ribosomal protein S7 gene (*AAEL009496*) was used as an endogenous reference, and the results were normalized using the virgin spermathecae control group.

### RNAi experiments

RNAi KD was done for eight selected transcripts putatively associated with energy metabolism (*Ae-92,048*), for the chitin associated transcripts (*Ae-187,521* and *Ae-88,956*), transcriptional regulation (*Ae-27,176*), hormonal regulation (*AeSigP- 4002*), enzymatic activity (*Ae-SigP-212,177*), antimicrobial activity (*AeSigP-109,183*), and ion homeostasis (*AeSigP-66,427*). Selection of transcripts for KD was based on their presumed role in maintaining sperm viability during storage within the spermathecae, as well as aspects of mosquito fitness, including survival, fecundity, oviposition or number of laid eggs, morphological characteristics of eggs, and number of eggs hatched (Additional file 1: Table S7). Before RNAi experiments, each predicted peptide sequence was aligned against homologous sequences present in *Ae. aegypti*, *Culex*, *Anopheles*, *Drosophila,* and *Homo sapiens*. Alignments and the primer amplification sites are shown in Figs. 1 and 2 in Additional file 4.

The selected genes were amplified by PCR reaction using the Taq 2X Master Mix (BioLabs® Inc.) kit, with 5 μM of the designed primers (Additional file 1: Table S5). The PCR product was separated on 1.5% agarose gel stained with ethidium bromide. The bands in the gel were cut, purified with ZymocleanTM Gel DNA Recovery Kit (Zymo Research), and sent for sequencing at Macrogen® (USA). The sequencing was analyzed using BLASTN against *Ae. aegypti* database (AaegL5 Liverpool strain) at VectorBase (version 5.2). For each identified coding sequence, we attributed a link to its profile on VectorBase (Additional file 5).

For dsRNA synthesis, primers were designed using the same sequence for the target sequences (Additional file 1: Table S5) with the addition of T7 promoter (5’GAATTAATACGACTCACTATAGGGAGA) using the MegaScript T7 transcription kit (Ambion, Austin, TX), according to the manufacturer’s protocol. After precipitation in ethanol/ammonium acetate solution, dsRNA was suspended in 1X PBS (0.1 M, pH = 7.6), and quantified using NanoDrop (Thermo Scientific) [66]. For dsRNA quality analysis, 6 μg of the samples were run in a 1.5% agarose stained with ethidium bromide. The dsRNA was stored at − 80 °C until nano injection. EGFP was used as a negative control for dsRNA [64], and dsEGFP microinjections were performed identically to targeted genes.

*Ae. aegypti* (Rockefeller) were obtained from the DIM, Johns Hopkins University and Departamento de Biologia Geral (DBG/UFV). Here, mosquito rearing was done as described in the section “Sample preparation and RNA sequencing.” Newly-emerged females were separated from males to prevent mating. For injection, virgin females were anesthetized on ice, placed over a glass slide covered with filter paper, and injected in the mesothorax with 69 nL of a 3 μg/μL solution of dsRNA in PBS [67, 68], using the Nanoject II Injector® (Drummond Scientific) at a rate of 46 nL/seg. For each gene being targeted with dsRNA, 500 females were used per group, with 300 separated for survival analysis, and 200 for gene expression (RT-PCR) and morphology analyses. After the injection, females were transferred to cages and fed with 10% sucrose solution ad libitum.

Considering that the rate of gene silencing is highly variable and dependent upon multiple factors, including transcript and protein turnover rates, a time course of transcript levels at 24, 48, 72, and 96 h post-injection was performed by RT-PCR. Total RNA was extracted from the spermathecae of 10 individuals per each group of dsRNA (eight transcripts) and used for 1st strand cDNA synthesis. RT-PCR was performed as describe elsewhere [67–69]. dsRNA-induced silencing started on day one post-injection, with the peak in KD effect being observed 3 days after injection (Additional file 2: Figure S2). From the RT-PCR results, a new round of dsRNA injection was devised. For genes highly expressed in virgin spermathecae and to assess gene silencing on pre-mating and mating events, females were injected on day one after emergence. In contrast, for genes highly expressed in inseminated females and to assess gene silencing on post-mating events, injections were done on day two after emergence. Regardless of how mosquitoes were divided between the two groups above, on day three after emergence all injected females were allowed to copulate for up to 24 h inside their respective cages. After mating/copulation (i.e., day four after emergence), all males were removed from the cages, and KD effects were assessed individually for each of the eight experimental groups (dsRNA-targeted genes). As a control group for virgin and inseminated spermathecae, 500 females were injected with dsEGFP. For this assay, the samples were also dissected in PBS as described above.

### Knockdown effects

Effects of dsRNA microinjections on the expression profiles of spermathecal genes were assessed by using duplicate pools of the spermathecae of ten females 2 days after injection, in the case of virgin females, or 2 days after mating, in the case of inseminated females. Mosquito dissection, RNA extraction, DNAse I treatment, and RT-PCR expression was performed as described in the previous section.

The survival analysis of the injected females was done on data collected from three independent replicates with 100 females each. Females were kept in plastic cages and fed with cotton soaked with 10% sugar solution ad libitum. To check the mating effect on female survival, the females were allowed to mate on day one after dsRNA injection for 24 h. After this period, males and females were separated. Dead females were counted and removed daily for 10 days to assess the effect of gene KD on mosquito survival. A schematic design of the phenotypic experiments is shown in Additional file 2: Figure S9.

To assess the effects of dsRNA injection on blood-feeding behavior, 100 females were separated into a cage and allowed to mate with males. After 24 h, the males were removed from the cage. The next day, all the females in the cage were allowed to blood-feed for 30 min on mice anesthetized with 10% ketamine hydrochloride (Agener União, Embu-Guaçu, São Paulo, Brazil) and 2% Xylazine hydrochloride (Ceva Santé Animale, Paulínia, São Paulo, Brazil) (diluted 1:4) (with mouse rotation every 10 min) in accordance with the UFV Ethics Committee (Protocol 561/2016). After the blood meal, the fully engorged females were sorted, and provided with 10% sugar solution ad libitum and used for fertility and fecundity analyses.

Two groups of 10 blood-fed females were individually transferred to 50 mL plastic tubes with filter paper soaked in 10 mL of distilled water and covered with fabric nets. These females were offered 10% sugar solution soaked in cotton ad libitum, for 4 days. After 4 days, the filter paper with the eggs was removed for posterior fecundity assays. Females that did not lay eggs were dissected, and their spermathecae and ovaries were photographed using a light microscope Olympus BX50 coupled to a camera, Moticam 580 at DBG/UFV. Images were compared to the control.

The eggs laid by the injected females from the previous experiment were counted, aligned, and stuck to a white tape and photographed. The egg length and total area were measured in the digital images using the ImagePlus software®. The measurements were performed twice to avoid any experimental error. We took these measurements to check how each dsRNA injection affects the egg phenotype and whether the changes in the egg phenotype affect the egg viability in comparison with the control.

The viability of eggs laid by the injected females was assessed using four pools of 100 eggs, each placed in plastic cups with 100 mL of distilled water and turtle food. Eggs were allowed to hatch for 2 days (enough time for all viable eggs to hatch) and the number of live larvae was counted (number of emerged larvae/100 eggs). Fertility was assessed as the number of hatched eggs from 100 eggs. Eggs were randomly sampled.

The spermathecal phenotype was also assessed following dsRNA injections. To this end, spermathecae collected from groups of 10 injected virgin and inseminated females were fixed in fixative solution (4% paraformaldehyde and 0.4% picric acid in PBS, pH = 7.3) for 2 h. Fixed samples were rinsed in PBS, dehydrated in an ascendant series of ethanol (70–100%), and embedded in a Historesin embedding kit (Leica, Heidelberger, Germany). Thin sections (4 μm thick) were stained with hematoxylin and eosin (HE) and dried. The stained sections (4 μm) were mounted with Eukitt® Quick-hardening mounting medium (Fluka, Darmstadt, Germany) and photographed under the light microscope Olympus BX50 coupled to a camera, Moticam 580 at the Departamento de Biologia Geral/UFV. Additionally, an assessment of sperm motility was performed to account for effects of the KD in the gametes within the spermathecal reservoir. On the third day after the blood feeding, five females of each dsRNA-injected group were randomly separated from the cage and dissected in PBS. The spermathecae of each female were transferred to cleaned glass slides (three spermathecae of each female/slide) with PBS and covered with cover slips. Sperm motility was visually inspected in freshly dissected spermathecae also using the Olympus BX50 at room temperature, classified as motile or non-motile compared with the control group (dsEGFP). For non-motile cases, reservoirs were gently mechanically disrupted, freeing the sperm. This part of the experiment was performed in duplicates for each dsRNA and recorded accordingly (Additional files 5, 6, 7, 8, 9 and 10 Movies S1 through S5).


**Additional file 6: Movie S1.** Ovary (not developed) of *Ae. aegypti* female injected with dsRNA targeting the Na^+^/Ca^2+^ seven days after the blood meal.



**Additional file 7: Movie S2.** Sperm motility inside of the reservoir one day after mating. Spermathecae of inseminated *Ae. aegypti* female controls injected with dsRNA targeting EGFP.



**Additional file 8: Movie S3.** Reduced sperm motility inside of the reservoir one day after mating. Spermatheca of inseminated *Ae. aegypti* female injected with dsRNA targeting the Na^+^/Ca^2+^.



**Additional file 9: Movie S4.** No sperm motility inside of the reservoir five days after a blood meal. Spermatheca of inseminated *Ae. aegypti* female injected with dsRNA targeting the Na^+^/Ca^2+^.



**Additional file 10: Movie S5.** Sperm their motility re-established once the reservoir is disrupted or broken. Spermathecae of inseminated *Ae. aegypti* female injected with dsRNA targeting the Na^+^/Ca^2+^ at day five after blood feeding.


### RNA in situ hybridization

The in situ hybridization was performed using the FISH Tag RNA Red Kit, with Alexa Fluor 594 dye (Life Technologies, Eugene, OR). For probe synthesis, 1 μg of the purified PCR product generated in the purification step of the dsRNA production (as described above for the RNAi experiments) was used, according to the manufacturer’s protocol. The spermathecae (virgin and inseminated) were dissected, fixed for 1 h, washed in distilled water, and incubated in 500 μL of labeling solution (10% probe solution in 1X PBS) overnight. The whole tissue was washed three times with PBS and stained with 4′, 6-diamidino-2-phenylindole in 0.5 g/mL solution (DAPI) (1:1000) (Sigma-Aldrich, St. Louis, MO) at 23 ± 2 °C for 1 h in the dark. It was then mounted in 50% sucrose solution and photographed under the fluorescence microscope, Olympus BX53 microscope, coupled to an Olympus DP 73 digital camera, using a WU filter (Laboratório de Sistemática Molecular, Departamento de Biologia Animal/UFV). As the control, we used the EGFP as template for the probe synthesis and checked it in both the virgin and the inseminated spermathecae.

### Statistical analysis

Data were analyzed using GraphPad Prism v.6 software (GraphPad Software, Inc., La Jolla California USA) and plotted as bar graphs. The data were tested for normal distribution using Shapiro-Wilk normality test with α = 0.05. Survival analysis was performed by the Kaplan-Meier method, and differences were detected by Log-rank (Mantel-Cox) test with α = 0.05. In order to compare either expression levels and knock down phenotypic effects between virgin and inseminated spermathecae, the comparative analysis was performed by one-way analysis of variance (ANOVA) and paired analysis with the two-tailed *t*-test with a 95% confidence interval and α = 0.05.

## Supplementary information

**Additional file 1.** Tables of comparative gene expression analyses between virgin and inseminated *Ae. aegypti* spermathecae. Results show summaries of DEG and respective functional annotations, selected transcripts used in RNAi experiments, and phenotypic assessments.

**Additional file 2.** Expression profiles and fitness effects from knockdown of selected transcripts including survival, oviposition rates, fecundity, and morphological effect on *Ae. aegypti* female ovaries.

**Additional file 3.** Histological sections of the spermathecae of *Ae. aegypti* spermathecae (dsRNA-injected females and control).

**Additional file 4.** Sequence and alignment information of eight selected transcripts targeted for RNAi. Primer sequences used for dsRNA synthesis as well as amplicon sizes for RT-PCR assessment of expression profiles are shown.

**Additional file 5.** Hyperlinked excel spreadsheet with reassembled coding sequences and reads mapped with RPKM>1 from *Ae. aegypti* virgin and inseminated spermathecomes.

## Data Availability

This project was registered at the National Center for Biotechnology Information (NCBI) under the accession BioProject ID PRJNA507773. BioSample accessions were SAMN10505526 and SAMN10505527. The reads are found under SRA accession PRJNA507773.

## Abbreviations

ANOVA: One-way analysis of variance
CDD: Conserved domain database
cDNA: Complementary DNA
DEG: Deferential expressed genes
dsRNA: Double-stranded RNA
EGFP: Enhanced green fluorescent protein
FDR: False discovery rate
FISH: Fluorescent in situ hybridization
KD: Knockdown
KEGG: Kyoto encyclopedia of genes and genomes
KOG: Eukaryotic orthologous groups
PBS: Phosphate buffered saline
Pfam: Protein family database
RefSeq: Reference sequence database
Repbase: Database of repetitive DNA
RNAi: *RNA interference*
RPKM: Reads per kilo base per million mapped reads
rRNA: Ribosomal RNA
RT-PCR: Real-time polymerase chain reaction
SMART: Simple modular architecture research tool
SRA: Sequence read archives
TPM: Transcripts per kilobase million
Vg: Vitellogenin
