# Supplementary material for: Transcriptional profiling and physiological roles of Aedes aegypti spermathecal-related genes
Source: BMC Genomics. 2020 Feb 10;21:143. doi: 10.1186/s12864-020-6543-y (PMC7011475; doi:10.1186/s12864-020-6543-y)
Supplement: Supplementary file 6 — Additional file 1. Tables of comparative gene expression analyses between virgin and inseminated Ae. aegypti spermathecae. Results show summaries of DEG and respective functional annotations, selected transcripts used in RNAi experiments, and phenotypic assessments. [file 12864_2020_6543_MOESM1_ESM.pdf]

Additional File 1

Table 1. Overall assessment of DEG in the *Ae. aegypti* spermatheca libraries. The libraries were compared to the whole female body and between spermathecae of virgin (Vir) and inseminated (Ins) females. *significant at 0.05 FDR from X^2 test with P-values adjusted with the Bonferroni correction for multiple testing.

Condition	CDS Number	Vir 8x > Ins	Ins 8 x > Vir
All CDS	21,612	276	368
CDS with V or I library having RPKM >1	14,786	241	315
Reads of spermatheca 2x larger than whole body library *	13,555	166	58
Reads of spermatheca 8x larger than whole body library *	661	111	25
Reads of spermatheca 16x larger than whole body library *	375	93	18
Reads of spermatheca 32x larger than whole body library *	373	93	15
Reads of spermatheca 64x larger than whole body library *	175	53	10

Table 2. Functional annotation based on DEG. The DEG were compared between virgin (Vir) and inseminated (Ins) of *Ae. aegypti* spermatheca libraries with RPKM> 1 and their ratio (proportion) among the different groups. N: number of contigs from each functional group, SE: variance of RPKM analysis.

Class	Average RPKM Virgin	SE	Average RPKM Ins	SE	Virg/Ins	N	Percent
Cytoskeletal	169.31	26.94	168.40	29.22	1.01	437	2.96
Oxidant metabolism/detoxification	28.66	4.65	31.48	4.43	0.91	248	1.68
Extracellular matrix/cell adhesion	79.75	10.46	54.35	12.08	1.47	366	2.48
Immunity	51.65	15.26	122.43	44.96	0.42	126	0.85
Metabolism	40.96	2.61	55.03	4.23	0.74	1398	9.45
Nuclear export	12.76	2.02	14.49	2.94	0.88	54	0.37
Nuclear regulation	21.75	3.94	22.41	4.29	0.97	483	3.27
Protein export machinery	43.31	4.81	44.62	4.49	0.97	488	3.30
Protein modification machinery	59.84	8.80	65.44	9.62	0.91	398	2.69
Proteasome machinery	30.31	3.03	34.09	3.63	0.89	306	2.07
Protein synthesis machinery	258.16	22.27	507.82	45.62	0.51	425	2.87
Secreted	121.17	21.18	123.83	16.30	0.98	2216	14.99
Signal transduction	32.62	2.72	33.62	3.59	0.97	1687	11.41
Storage	102.68	36.11	193.28	71.89	0.53	21	0.14
Transposable element	13.02	1.76	13.03	1.76	1.00	711	4.81
Transcription factor	17.98	2.49	29.27	9.90	0.61	357	2.41
Transcription machinery	47.60	14.26	39.59	7.68	1.20	911	6.16
Transporters/storage	39.94	4.82	53.27	6.70	0.75	463	3.13
Unknown, conserved	221.24	49.01	144.76	29.87	1.53	2744	18.56
Unknown	339.12	92.68	210.76	50.13	1.61	934	6.32
Viral	27.87	14.58	113.85	61.82	0.24	13	0.09

Table 3. Functional annotation based on DEG. Annotation of DEG with at least eightfold expression in virgin (Vir) than inseminated (Ins) spermathecae. N: number of contigs from each functional group, SE: variance of RPKM analysis.

Class	Average RPKM (Vir)	SE	Average RPKM (Ins)	SE	Vir/Ins	N	Percent
Cytoskeletal	386.97	0.00	15.43	0.00	25.09	1	0.90
Extracellular matrix/cell adhesion	203.64	48.76	7.78	2.26	26.17	43	38.74
Metabolism	43.54	15.98	3.64	1.90	11.96	9	8.11
Nuclear regulation	486.02	0.00	25.09	0.00	19.37	1	0.90
Protein modification machinery	20.21	15.23	0.88	0.73	22.91	5	4.50
Secreted	967.22	645.31	47.50	30.02	20.36	27	24.32
Signal transduction	35.83	11.01	2.34	0.77	15.34	8	7.21
Transcription machinery	46.21	36.70	0.73	0.55	63.33	3	2.70
Transporters/storage	260.73	0.00	11.29	0.00	23.08	1	0.90
Unknown, conserved	15.68	4.69	1.02	0.41	15.35	8	7.21
Unknown	57.21	17.26	2.78	0.90	20.57	5	4.50
Total						111	100

Table 4. Functional annotation based on DEG. Annotation of DEG with at least eightfold expression in inseminated (Ins) than in virgin (Vir) spermathecae between the different samples. N: number of contigs from each functional group, SE: variance of RPKM analysis.

Class	Average RPKM (Vir)	SE	Average RPKM (Ins)	SE	Ins/Vir	N	Percent
Oxidant metabolism/detoxification	26.81	0.00	352.90	0.00	13.12	1	4.00
Extracellular matrix/cell adhesion	0.00	0.00	2.86	0.00	28.64	1	4.00
Immunity	0.08	0.00	5.99	0.00	32.87	1	4.00
Metabolism	4.22	2.49	304.39	245.00	70.47	3	12.00
Protein modification machinery	1.42	0.00	29.55	0.00	19.41	1	4.00
Secreted	66.39	35.96	1,357.85	986.52	20.42	11	44.00
Signal transduction	46.98	38.38	445.46	293.03	9.46	2	8.00
Transposable element	7.88	7.13	104.25	94.25	13.06	2	8.00
Unknown, conserved	181.92	148.53	2,119.84	1,727.68	11.65	3	12.00
Total						25	100

Table 5. Transcripts identified from the *Ae. aegypti* spermatheca and selected for RNAi knockdown and DEG analyses. Columns from left to right are gene number based on NCBI accession number (gene); putative function (related function); Sequence direction and primer pair used for analysis of expression profiles (sequence direction and primer sequence); and presence of transcript per each of the two libraries analyzed as determined by the abundance of transcript in the RNAseq assessment (*in silico* expression value).

Gene ID number	Putative function	Primer sequence	*In silico* expression value	
			Virgin	Inseminated
Ae-92048	Glucose dehydrogenase (Gld)	**F – CAAAGTGCTGAGATGGCTGGACT**<br>**R - ATCGTCGGGATATGGCGAAACAG**	**57.491**	**0**
Ae-187521	Chitin bind 4 (ChtB4)	**F - TGCTGGAAAGTCACATTCTCAATCA**<br>**R - GCATCCTGTCCCGGTTGAATACC**	**248.575**	**0.00368**
Ae-27176	Atrophin-1 protein (Atro-1)	**F - GCAAGGAATGGTGCTTCAAGCTG**<br>**R - TGTAGAAGGGCAACTTGCGAGTC**	**38.853**	**0.0219**
AeSigP-4002	DHR4 ligand (DHR4)	**F - ATGTCACAATCCCCACAGAACGG**<br>**R - GATTTCTGGTTGTTCGCTTGGCC**	**1042.263**	**0.000822**
Ae-88956	N-acetylgalactosaminyl transferase 6 (GALNT6)	**F - AAGCAACGTCTGGCAAGTGTCGG**<br>**R - TGGCAGCGAATCGAGAGTTGTAC**	**24.046**	**0.0308**
AeSigP-66427	Chitin-binding type-2 (ChtBD2)	**F - ACGGTGTTGGTGGTTTTCTCGAT**<br>**R - CCTTGGTTGTAGGCACCATCCAA**	**0.0044**	**16.733**
AeSigP-109183	Kazal type serine protease inhibitor (KSPI)	**F - AATCAGTGCATTGCTTTTCGC**<br>**R - AGACACTGAACAGCTTTTCCCGA**	**0.0068**	**96.478**
AeSigP-212177	Na^+/Ca^{2+} exchanger protein (Na^+/Ca^{2+})	**F - CACGTGAAACTTGATTGGTGGCC**<br>**R - CACCGATCCACGCTATGCACATA**	**0.0190**	**51.690**

Table 6. Relative expression of selected genes in the spermathecae of virgin and inseminated *Ae. aegypti* females before and after dsRNA injections. Relative expression was calculated from RT-PCR results and are based on fold change. For paired comparison Tukey's multiple comparisons test (α=0.05) was used. *Gld* (P>0.005); *ChtB4* (P=0.6342); *Atro-1* (P>0.005); *DHR4* (*AeSigP-4002*) (P>0.005); *GALNT6* (P=0.2378); *ChtBD2* (P=0.3739); *KSPI* (P=0.2996); *Na^+/Ca^{2+}* (P>0.005). The graphical representation is shown in Figure 1, Additional File 2.

Gene ID number	Putative function	Before injections		After injections	
		Virgin	Inseminated	Virgin	Inseminated
AAEL009496	S7 ribossomal subunit (S7)	**1.0**	**1.0**	**1.0**	**1.0**
Ae-92048	Glucose dehydrogenase (Gld)	**1.003**	**0**	**0**	**0**
Ae-187521	Chitin bind 4 (ChtB4)	**0.155**	**0**	**0.0366**	**0.0633**
Ae-27176	Atrophin-1 protein (Atro-1)	**0.6**	**0.102**	**0**	**0**
AeSigP-4002	DHR4 ligand (DHR4)	**1.138**	**0**	**0**	**0**
Ae-88956	N-acetylgalactosaminyl transferase 6 (GALNT6)	**15.16**	**4.233**	**0**	**0.0166**
AeSigP-66427	Chitin-binding type-2 (ChtBD2)	**0.225**	**0.7**	**0**	**0.03**
AeSigP-109183	Kazal type serine protease inhibitor (KSPI)	**0.0975**	**5.58**	**0**	**0.0933**
AeSigP-212177	Na^+/Ca^{2+} exchanger protein (Na^+/Ca^{2+})	**0.1475**	**3.69**	**0**	**0**

Table 7. Phenotypic analysis in *Ae. aegypti* females after injection of dsRNA. The values represent mean ± SD.

Gene	Related function	Phenotypic analysis - characteristics					
		Blood meal (number of females)	Oviposition (number of females)	Survival after oviposition (number of females)	Fertility (number of eggs laid)	Egg length (mm)	Egg area (mm^2)
**Virgin control**	**dsEGFP**	8.66±0.57	7.5±0.70	7.5±0.70	69.7±10.60	0.77±0.03	0.16±0.02
**Ae-92048**	Glucose dehydrogenase	6.66±0.57	8±0.01	8±0.01	49.6±27.36	0.82±0.02	0.17±0.01
**Ae-187521**	Chitin bind 4	8.33±0.57	5.5±0.70	6.5±0.70	64.5±20.87	0.62±0.04	0.11±0.01
**Ae-27176**	Atrophil-1 protein	8.33±0.57	7±0.01	7.5±0.70	69.5±15.27	0.72±0.02	0.13±0.01
**AeSigP-4002**	DHR4 ligand	9.33±1.15	6.5±0.70	6.5±0.70	51.4±29.12	0.51±0.03	0.07±0.01
**AeSigP-212177**	N-acetylgalactosaminyl transferase	9.33±0.57	5±0.01	5.5±0.70	76.6±20.52	0.50±0.07	0.07±0.01
**Mated control**	**dsEGFP**	7.33±0.57	5±0.01	5.5±0.70	63±4.52	0.54±0.02	0.08±0.01
**Ae-88956**	Chitin-binding domain type 2	6.66±0.57	6±1.41	5.5±0.70	48.7±14.41	0.52±0.01	0.07±0.02
**AeSigP-109183**	Kazal type serine protease inhibitor	8.33±0.57	5.5±0.70	5±0.01	75.8±12.56	0.53±0.03	0.07±0.01
**AeSigP-66427**	Na^+/Ca^{2+} exchanger protein	5.33±0.57		5.5±0.70			
