# Supplementary material for: Transcriptional profiling and physiological roles of Aedes aegypti spermathecal-related genes
Source: BMC Genomics. 2020 Feb 10;21:143. doi: 10.1186/s12864-020-6543-y (PMC7011475; doi:10.1186/s12864-020-6543-y)
Supplement: Supplementary file 7 — Additional file 2. Expression profiles and fitness effects from knockdown of selected transcripts including survival, oviposition rates, fecundity, and morphological effect on Ae. aegypti female ovaries. [file 12864_2020_6543_MOESM2_ESM.pdf]

Additional File 2

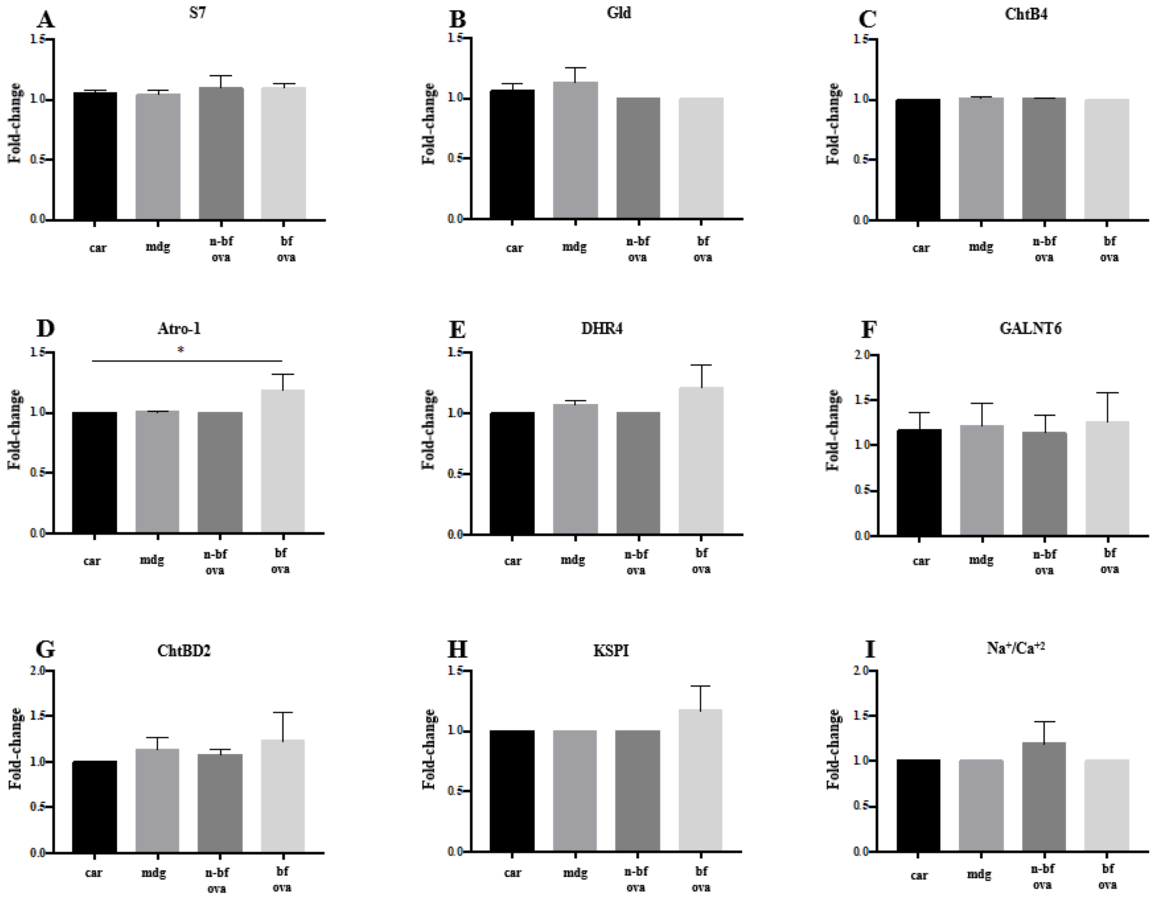

Figure 1: Differential tissue-specific expression of selected genes in *Ae. aegypti* females. Relative expression by RT-PCR of a pool of 10 females for the selected genes in carcass (car), midgut (mdg) and ovaries from virgin non-blood-fed (n-bf ova) and inseminated blood-fed (bf ova) *Ae. aegypti* females. Bars show the fold-change of each sample normalized to ribosomal gene S7. Reactions were done in triplicate using two biological replicates. Statistical analyses were performed using one-way ANOVA and Tukey's multiple comparison test (α=0.05). For each transcript, the respective statistical values were determined for F, R^2 and P. **A:** *S7* (F=0.7268; R^2=0.2142; P=0.5641), **B:** *Gld* (F=2.429; R^2=0.4767; P=0.1404), **C:** *ChtB4* (F=1.286; R^2=0.3253; P=0.3437), **D:** *Atro-1* (F=5.915; R^2=0.6893; P=0.0199; *P=0.0349), **E:** *DHR4* (F=3.048; R^2=0.5334; P=0.0922), **F:** *GALNT6* (F=0.1393; R^2=0.04967; P=0.9336), **G:** *ChtBD2* (F=0.8578; R^2=0.2434; P=0.5010), **H:** *KSPI* (F=2.036; R^2=0.4329; P=0.1875), **I:** *Na^+/Ca^{2+}* (F=1.773; R^2=0.3994; P=0.2298). Transversal bars represent the errors in each studied group.

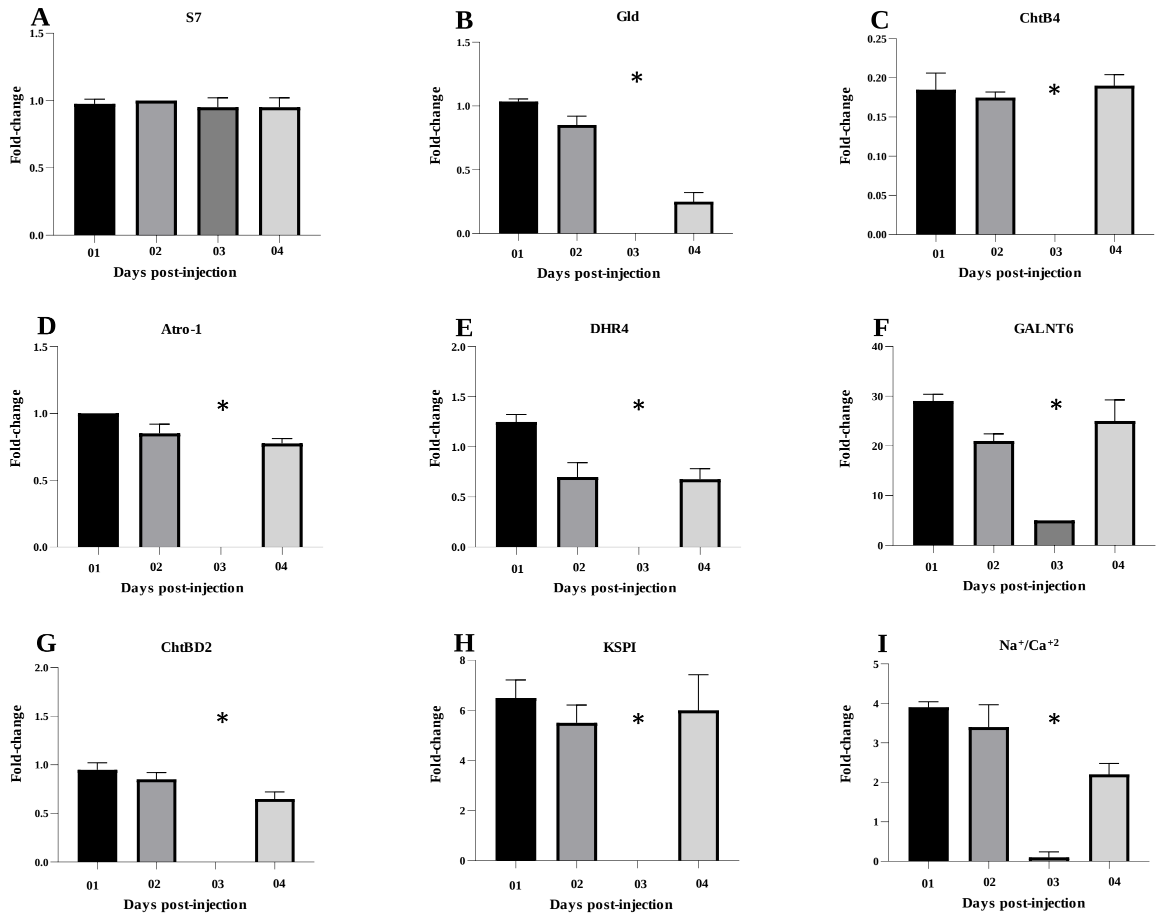

Figure 2. Relative expression of selected genes in the spermathecae of virgin and inseminated *Ae. aegypti* females after dsRNA injections. Relative expression for each gene was calculated for days 1 through 4 after injection. RT-PCR results represent fold change. For paired comparison Tukey's multiple comparisons test (α=0.05) was used. **A:** *S7* (P=0.7567); **B:** *Gld* (P<0.0001); **C:** *ChtB4* (P=0.003); **D:** *Atro-1* (P<0.0001); **E:** *DHR4* (P=0.0009); **F:** *GALTN6* (P=0.0019); **G:** *ChtBD2* (P=0.0003); **H:** *KSPI* (P=0.0496); **I:** Na^+/Ca^{2+} (P=0.0012).

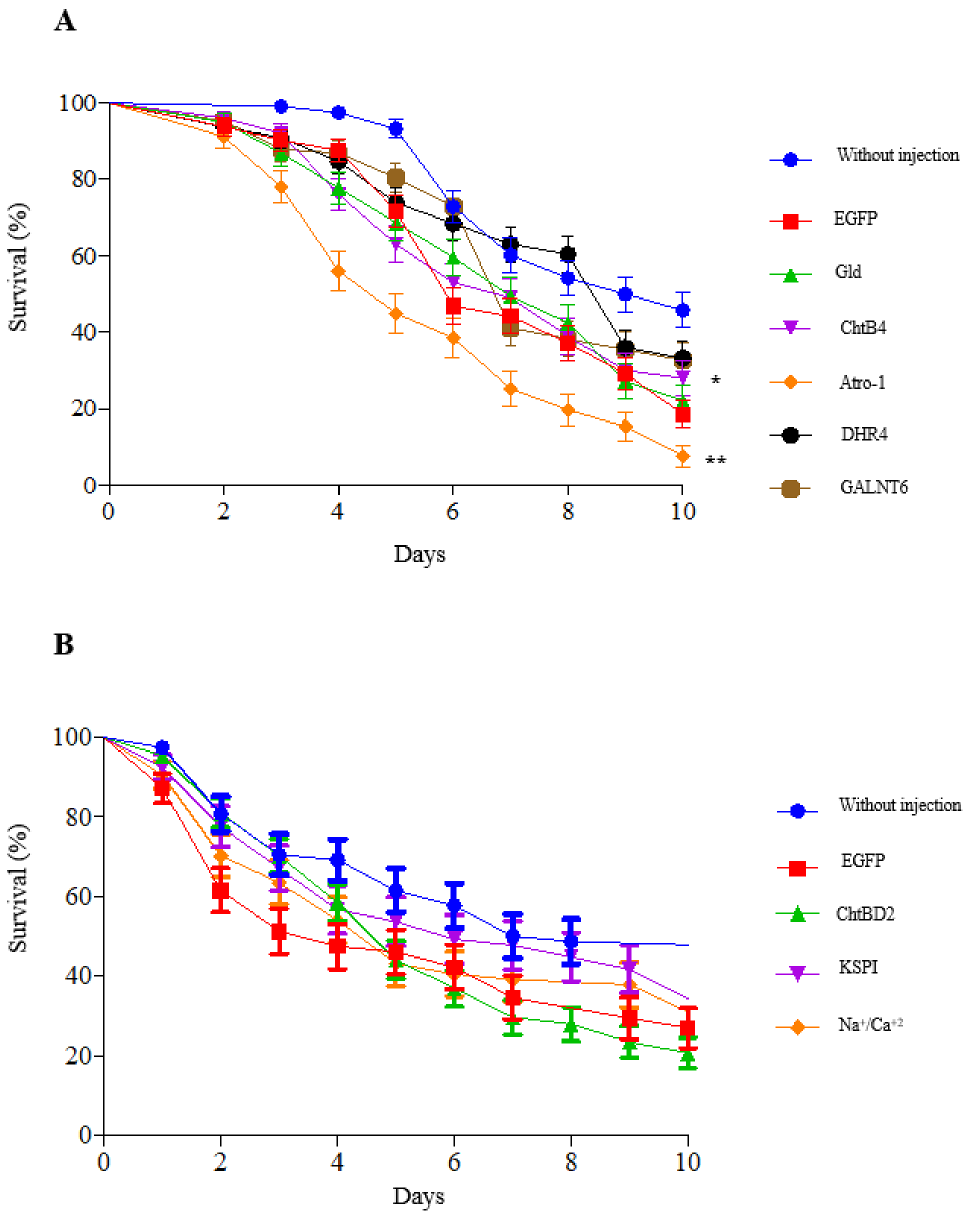

Figure 3. Survival of *Ae. aegypti* females (virgin and inseminated) after injection of dsRNA. Each treatment was performed on 100 females, and survival recorded. Differences between each targeted gene and the dsEGFP control were calculated by the Log-rank test (α=0.05). **A:** Injections for genes highly expressed in virgin spermathecae (X^2=13.84, P=0.0166, *P=0.0364; **P=0.0109); **B:** Injections for genes highly expressed in inseminated spermathecae (X^2=2.421, P=0.1197). Bars represent the variance.

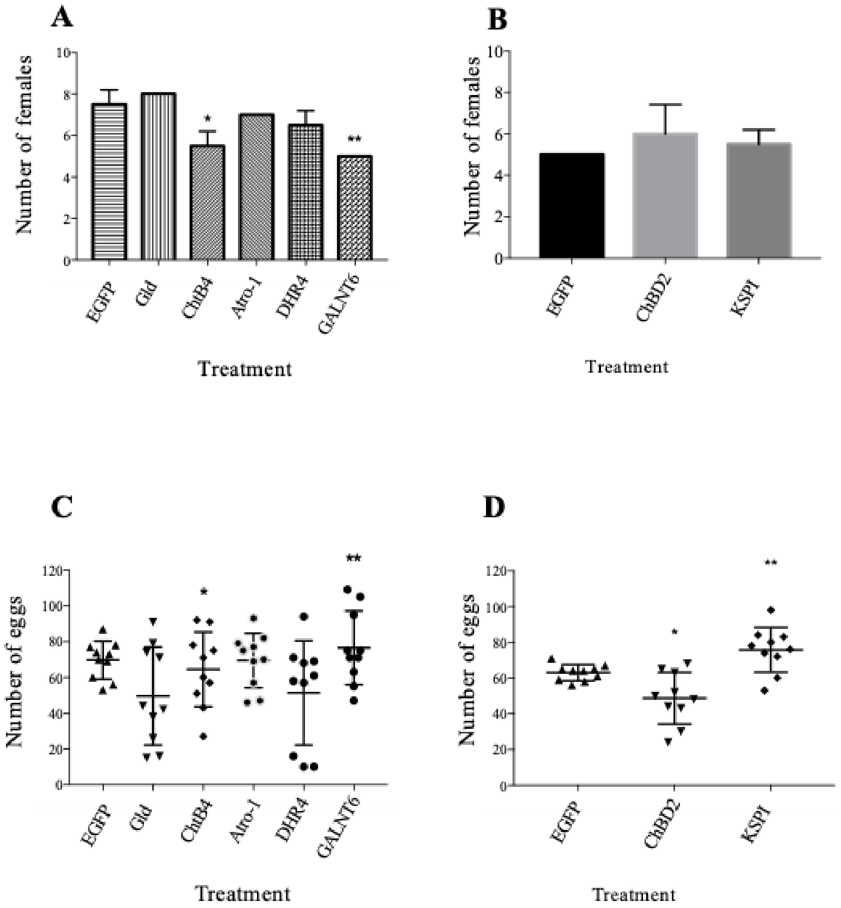

Figure 4. Oviposition and fertility rates. Effect of dsRNA injections on the oviposition, assessed as the number of *Ae. aegypti* females that laid eggs (A, B), and fertility, assessed as the number of eggs laid (C, D). Values for each gene were compared with the control (EGFP) by one-way ANOVA and Tukey's multiple comparisons test (α=0.05). For each treatment, the respective statistical values were determined for F, R^2 and P. **A** and **C** represent the number of females that laid eggs after dsRNA injections and following blood feeding (A), and their corresponding fertility (F=10.73, R^2=0.8994, P=0.0059; *P=0.00489, **P=0.0179). Targeted genes (X-axis) were highly expressed in virgin spermathecae. **B** and **D** represent the number of females that laid eggs after dsRNA injections and following blood feeding (F=0.6, R^2=0.2857, P=0.6037), and their corresponding fertility (F=14.28, R^2=0.514, P<0.0001; *P=0.0235, **P=0.0455. Targeted genes (X-axis) were highly expressed in inseminated spermathecae. Transversal bars in C and D represent the average.

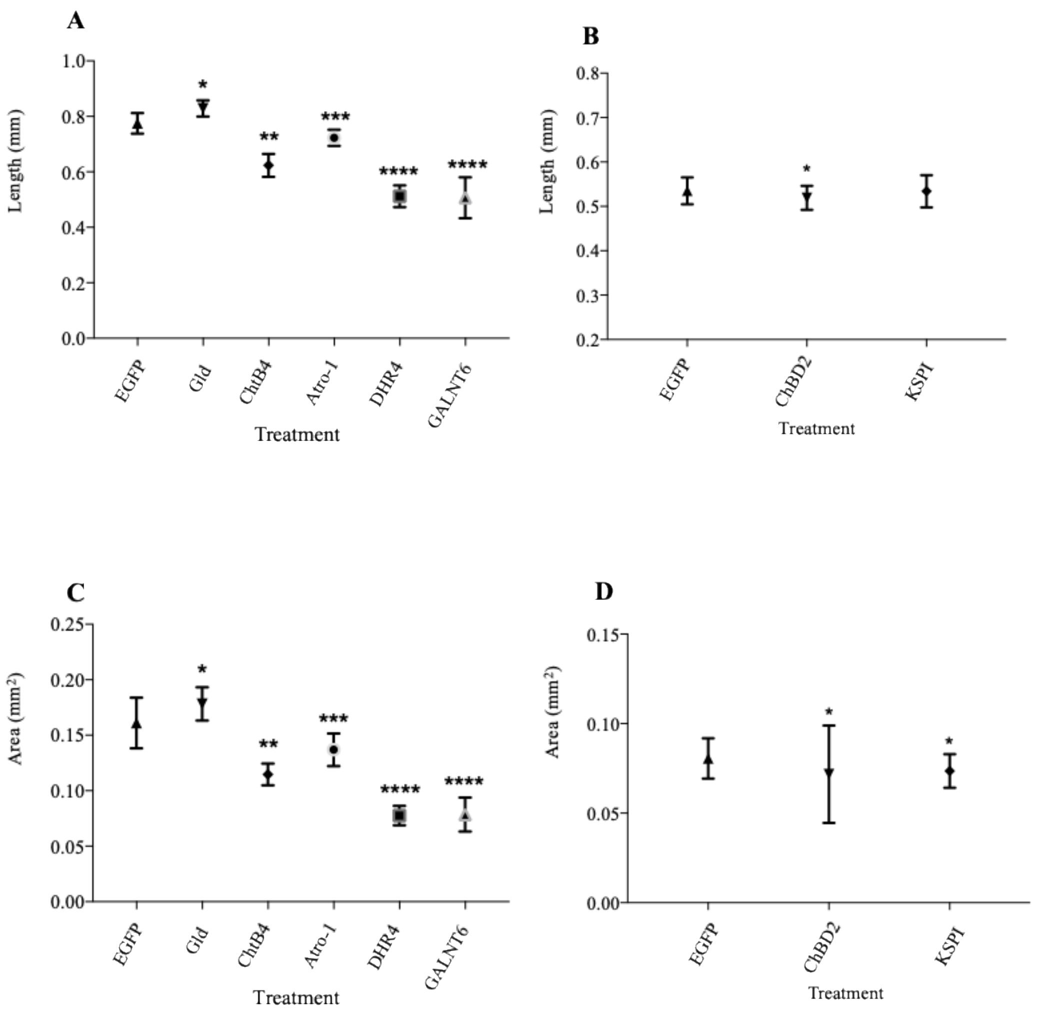

Figure 5. Morphometry of eggs laid by *Ae. aegypti* females injected with dsRNA for different target genes. Values for each gene were compared with the control (EGFP) by one-way ANOVA and Tukey's multiple comparisons test (α=0.05). For each treatment, the respective statistical values were determined for F, R^2 and P. **A:** Length of eggs produced by females injected with dsRNA for genes highly expressed in virgin spermathecae (F=963, R^2=0.8882, P<0.0001; *, **, ***, ****P<0.0001). **B**: Length of eggs produced by females injected with dsRNA for genes highly expressed in inseminated spermathecae (F=8.213, R^2=0.05241, P=0.0003; *P=0.0001). **C**: Area of eggs produced by females injected with dsRNA for genes highly expressed in virgin spermathecae (F=744.1, R^2=0.8409, P<0.0001; *, **, ***, ****P<0.0001). **D**: Area of eggs produced by females injected with dsRNA for genes highly expressed in inseminated spermathecae (F=6.803, R^2=0.0438, P=0.0013; *P=0.0163).

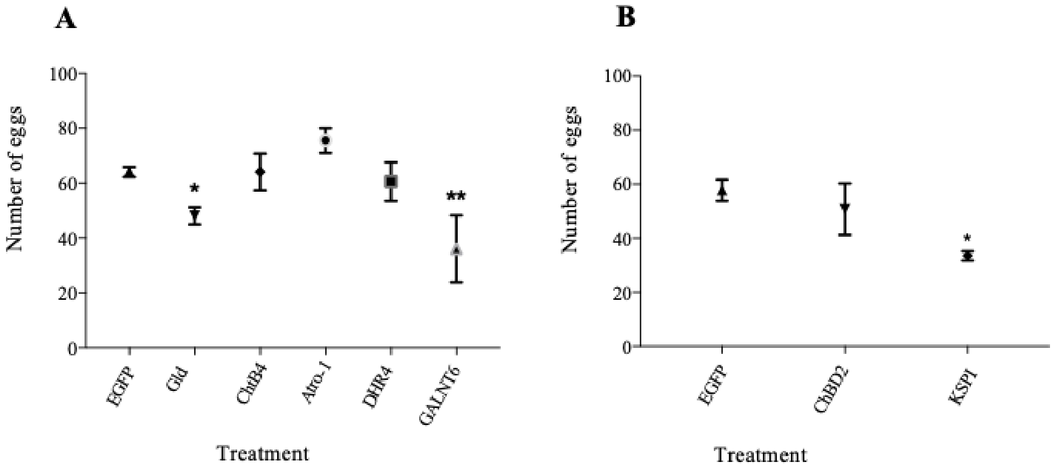

Figure 6. Effect of dsRNA injections on the fecundity of *Ae. aegypti* females. Values for each targeted gene were compared with dsEGFP control by one-way ANOVA and Tukey's multiple comparisons test (α=0.05). For each treatment, the respective statistical values were determined for F, R^2 and P. **A:** Egg-hatching of females injected with dsRNA for genes highly expressed in virgin spermathecae (F=16.84, R^2= 0.8239, P<0.0001; *P=0.0365, **P=0.0002). **B:** Egg-hatching of females injected with dsRNA for genes highly expressed in inseminated spermathecae (F=17.18, R^2=0.7924, P=0.0008; *P=0.0008).

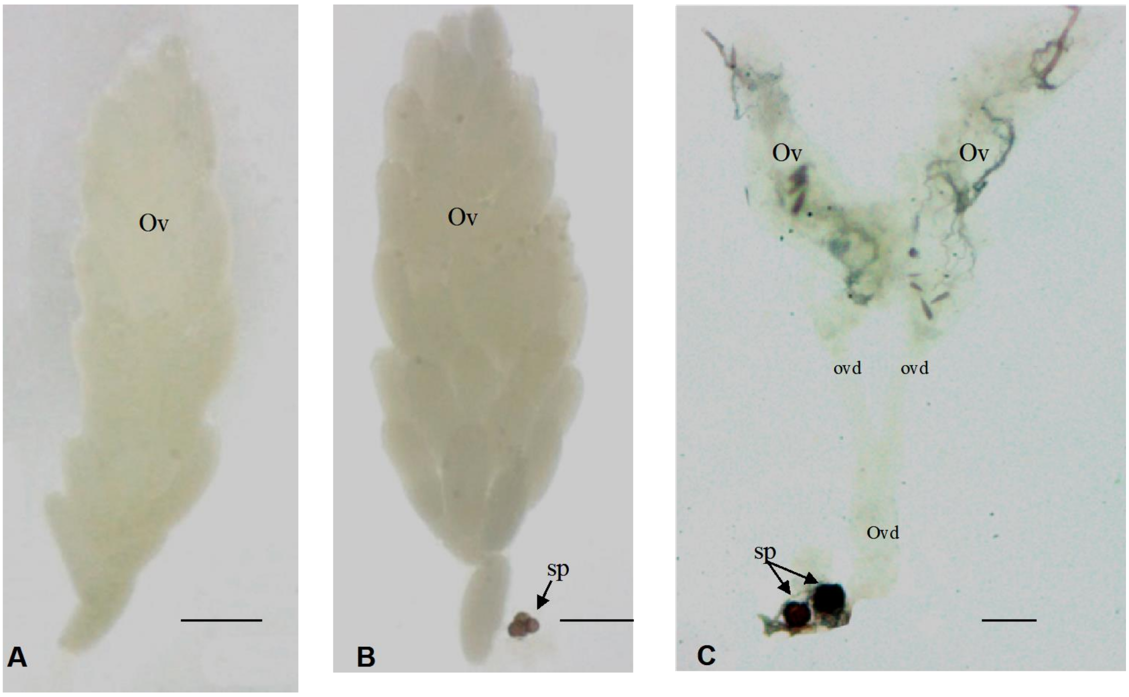

Figure 7. Morphology of the ovaries in *Ae. aegypti* females three days after blood-feeding. A: Virgin females injected with dsEGFP control. B: Inseminated females injected with dsEGFP. C: Inseminated females injected with dsRNA targeting *Na^{+}/Ca^{2+}*. (Ov): Ovary; (ovd): lateral oviducts; (Ovd): common oviduct; (sp): spermathecae. Bar: 1 mm.

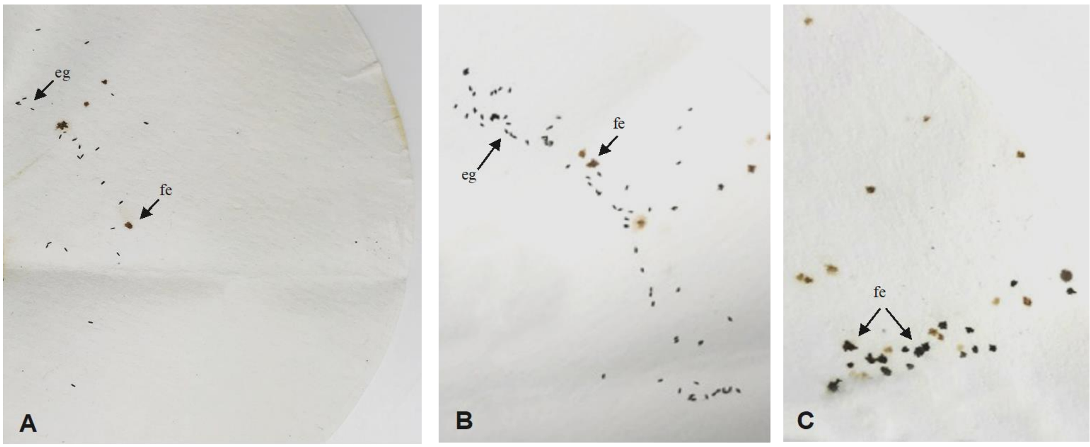

Figure 8. Filter papers offered to females to lay eggs five days after the blood meal. A: Oviposition of virgin females injected with dsEGFP. **B:** Oviposition of inseminated females injected with dsEGFP. **C:** Inseminated females injected with dsRNA targeting Na^{+}/Ca^{2}. (eg): eggs; (fe): pigmented feces resulted from blood digestion.

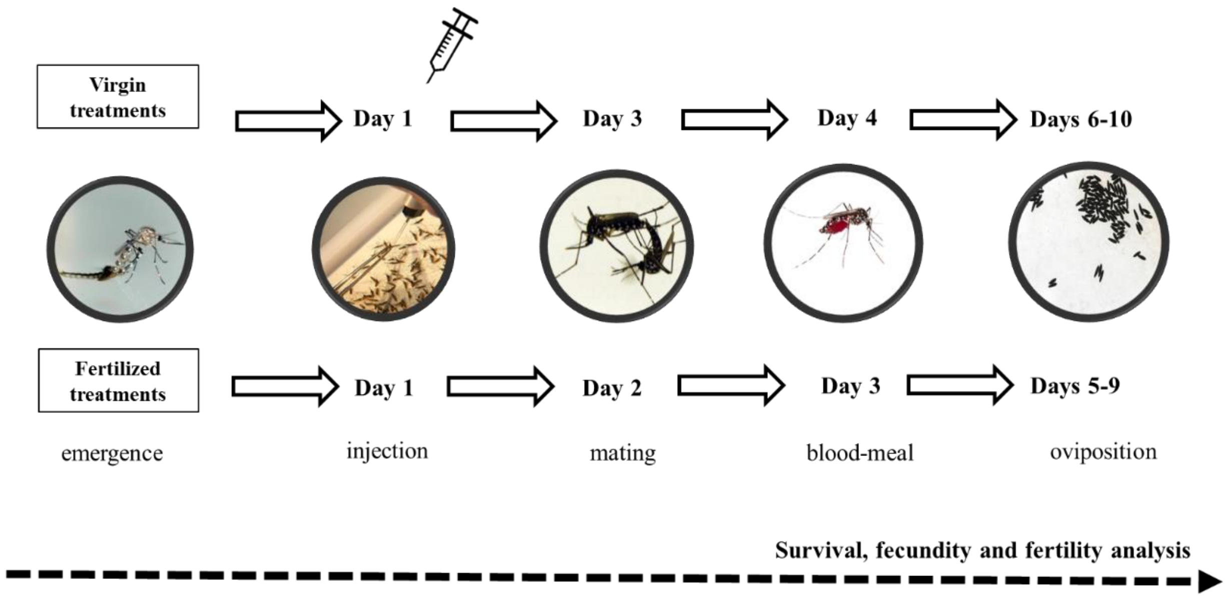

Figure 9. Scheme representative of the experimental design for dsRNA injections followed by the phenotypic analysis.
