# Supplementary material for: Transcriptional profiling and physiological roles of Aedes aegypti spermathecal-related genes
Source: BMC Genomics. 2020 Feb 10;21:143. doi: 10.1186/s12864-020-6543-y (PMC7011475; doi:10.1186/s12864-020-6543-y)
Supplement: Supplementary file 8 — Additional file 3. Histological sections of the spermathecae of Ae. aegypti spermathecae (dsRNA-injected females and control). [file 12864_2020_6543_MOESM3_ESM.pdf]

Additional File 3

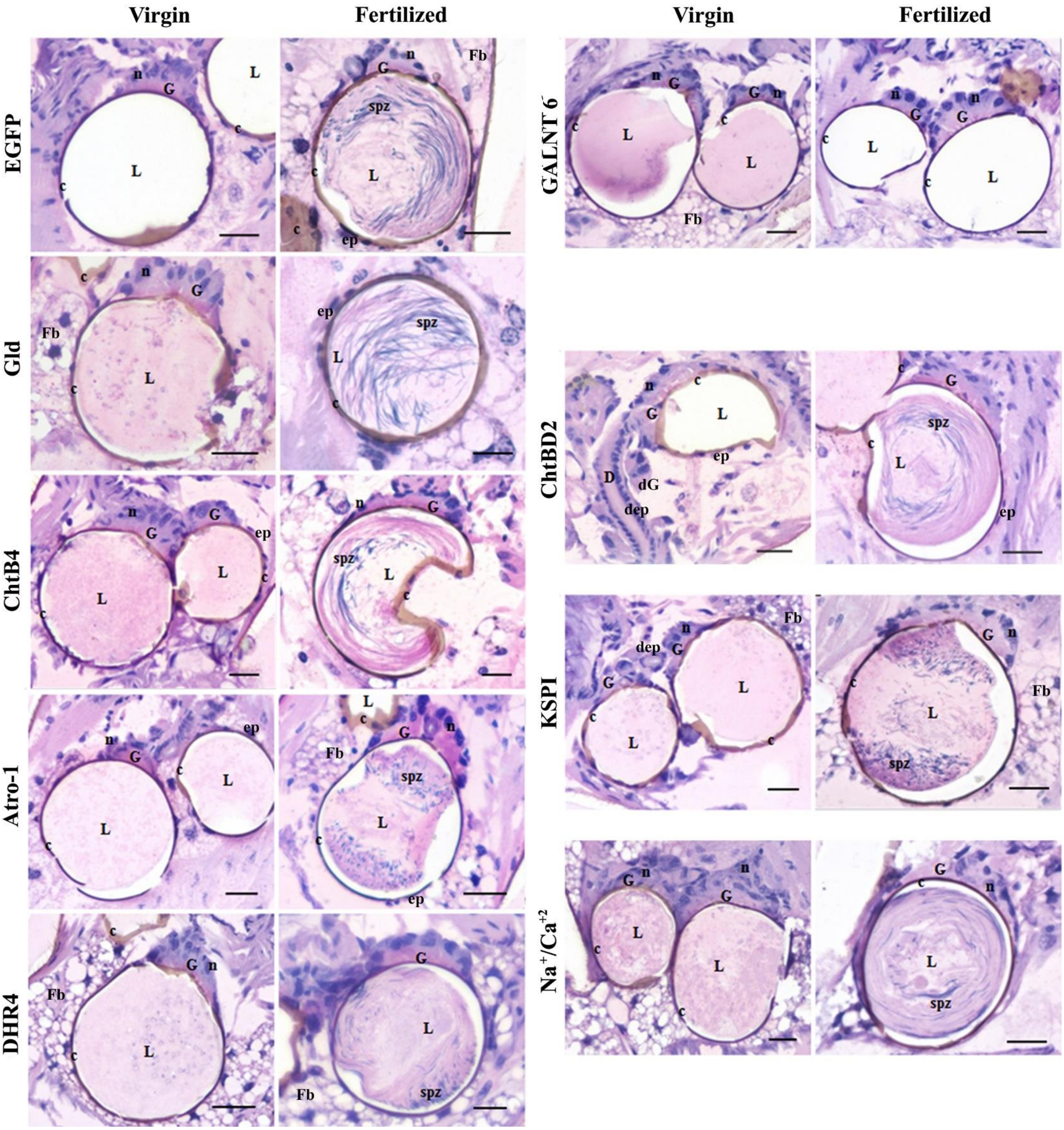

Histological sections stained with HE of spermathecae of *Ae. aegypti* (virgin and inseminated females) after dsRNA injection for *EGFP, Gld, ChtB4, Atro-1, DHR4, ChtBD2, Na^{+}/Ca^{2+}, KSPI,* and *GALNT6.* (c) reservoir cuticle, (ep) flattened reservoir epithelium, (D) spermathecal duct, (G) spermathecal gland, (dG) duct gland cell, (dep) epithelium of spermathecal duct, (Fb) fat body, (L) reservoir lumen, (n) nucleus of gland cell, (spz) spermatozoa. Specific KD effects can be compared with dsEGFP injected (control). In the inseminated spermathecae of all individuals, the sperm is organized circularly within the reservoir lumen being arranged parallel to each other. Spermathecal parts are describe in Fig 1 of the body of the manuscript. Bar: 10 μm.
