# Supplementary material for: Transcriptional profiling and physiological roles of Aedes aegypti spermathecal-related genes
Source: BMC Genomics. 2020 Feb 10;21:143. doi: 10.1186/s12864-020-6543-y (PMC7011475; doi:10.1186/s12864-020-6543-y)
Supplement: Supplementary file 9 — Additional file 4. Sequence and alignment information of eight selected transcripts targeted for RNAi. Primer sequences used for dsRNA synthesis as well as amplicon sizes for RT-PCR assessment of expression profiles are shown. [file 12864_2020_6543_MOESM4_ESM.pdf]

Additional File 4

Gld

```
   1 M  K  L  S  V  W  T  S  L  V  L  L  V  C  L  S  G  P  C  I  A  T  V  V  R  K  L  T  L  L
   1 ATGAAGCTCAGCGTTTGGACTAGTCTCGTGTTGCTTGTTTGCCTTTCTGGACCATGTATTGCCACGGTTGTGCGAAAATTGACCCTTCTA
  31 G  P  Y  G  N  H  T  L  G  D  Y  V  D  F  S  S  A  W  G  L  D  Y  G  N  P  N  P  K  I  R
  91 GGTCCTTACGGAAATCATACACTCGGAGACTACGTTGATTTCAGCAGTGCGTGGGGATTGGACTATGGTAACCCCAATCCGAAAATTCGA
  61 K  S  Y  D  F  I  V  V  G  A  G  P  A  G  C  S  V  A  N  H  L  S  E  N  P  D  V  T  V  L
 181 AAATCTTACGACTTCATCGTCGTTGGAGCTGGACCGGCAGGCTGCTCTGTGGCGAACCATTTATCTGAAAACCCTGACGTGACAGTGCTT
  91 L  L  E  L  G  K  A  E  I  A  P  T  Q  D  I  P  S  G  F  L  F  Q  T  A  T  D  Y  N  F  G
 271 CTTCTTGAGCTTGGCAAAGCGGAAATCGCTCCAACCCAAGATATTCCTAGTGGATTTCTGTTTCAAACTGCAACGGATTACAACTTTGGG
 121 Y  L  S  Q  P  Q  T  K  G  C  Q  G  L  I  N  K  Q  C  A  F  H  H  G  R  G  L  G  G  S  T
 361 TACTTGAGTCAGCCGCAGACCAAAGGATGTCAAGGTCTCATCAACAAACAGTGCGCGTTTCATCACGGTCGTGGTCTTGGAGGTTCGACT
 151 I  I  N  N  M  I  Y  T  R  G  N  W  R  D  F  D  G  W  N  A  S  G  N  P  G  W  S  Y  R  E
 451 ATAATAAACAACATGATCTACACCAGAGGAAACTGGCGAGACTTTGATGGTTGGAATGCGTCGGGAAATCCTGGATGGAGCTACCGCGAA
 181 V  L  P  Y  F  I  K  A  E  N  A  N  L  R  D  F  G  N  N  G  F  H  G  K  D  G  Y  L  S  V
 541 GTACTTCCCTATTTCATCAAGGCAGAGAATGCGAATCTGAGAGATTTCGGAAACAATGGCTTTCACGGGAAGGACGGTTATTTGTCGGTT
 631                                           CAAAGTGCTGAGATGGCTGGACT 3' FW
 211 E  D  I  P  Y  R  S  R  L  A  S  T  F  I  Q  S  A  E  M  A  G  L  P  Y  I  D  Y  N  T  M
 631 GAAGATATACCCTATCGATCTCGTTTGGCATCAACCTTTATCCAAAGTGCTGAGATGGCTGGACTTCCTTACATTGATTACAACACTATG
 241 D  Q  L  G  S  S  Y  I  Q  S  N  T  K  R  G  V  R  W  T  A  A  R  A  L  L  N  P  I  R  N
 721 GATCAACTGGGATCTTCTTACATTCAATCGAATACTAAAAGAGGCGTACGCTGGACTGCAGCTAGAGCTTTGTTGAATCCCATAAGAAAT
 271 R  K  N  L  H  V  L  T  R  A  W  A  T  K  V  L  I  D  K  S  K  V  A  Y  G  V  V  Y  T  R
 811 CGAAAAAATCTCCATGTATTAACTCGAGCTTGGGCCACAAAAGTGCTAATCGATAAATCAAAAGTTGCTTATGGCGTGGTATACACAAGG
 301 D  K  K  T  Y  T  V  K  A  K  R  E  V  I  L  S  A  G  A  F  G  S  A  K  L  L  M  L  S  G
 901 GATAAGAAAACATACACCGTGAAGGCCAAACGAGAAGTAATCCTCTCTGCTGGGGCATTTGGAAGCGCTAAGTTACTAATGCTGTCAGGA
 331 V  G  P  K  S  H  L  Q  D  L  G  I  D  V  I  K  D  L  P  V  G  E  T  L  Y  E  H  P  G  V
 991 GTCGGTCCAAAAAGTCACCTCCAAGATCTTGGTATTGACGTTATCAAAGATCTGCCAGTTGGTGAAACTCTCTACGAACATCCAGGTGTC
 361 L  G  P  V  F  L  V  T  K  P  I  D  N  N  I  N  F  E  S  L  I  T  L  P  N  I  I  K  Y  L
1081 CTAGGTCCAGTATTTCTAGTCACTAAACCAATTGATAACAACATAAACTTCGAAAGCCTCATTACGCTTCCAAACATTATCAAATATTTA
1171                                                           GACAAAGCGGTATAGGGCTGCTA 5' RV
 391 F  G  Q  G  P  F  T  S  A  F  T  E  T  V  G  Y  V  K  S  P  V  S  P  Y  P  D  D  P  D  W
1171 TTTGGCCAAGGACCATTTACTTCAGCATTCACAGAAACAGTGGGATACGTCAAATCACCTGTTTCGCCATATCCCGACGATCCAGACTGG
 421 P  D  L  E  I  I  L  S  A  L  Q  I  G  D  D  P  T  T  A  G  R  T  Y  F  R  V  N  D  G  I
1261 CCTGATCTGGAAATAATTTTATCGGCCCTTCAGATTGGAGATGACCCAACCACTGCTGGAAGAACCTATTTCAGAGTCAACGATGGAATA
 451 R  E  S  Y  F  R  P  L  F  H  T  R  A  F  M  Y  L  P  L  L  M  H  S  R  S  K  G  S  I  K
1351 CGAGAGTCGTATTTCCGACCTCTCTTCCATACCCGTGCCTTCATGTATCTGCCACTCCTTATGCATAGCAGATCAAAGGGATCGATCAAG
 481 L  K  S  T  N  P  Y  D  H  P  L  F  N  Y  T  Y  F  D  D  D  R  D  L  Q  A  L  V  Y  A  I
1441 TTGAAGTCCACCAATCCGTACGATCATCCACTGTTCAACTATACGTACTTTGATGATGATCGTGACCTGCAAGCGTTGGTGTATGCCATC
 511 K  E  A  I  R  I  T  G  Q  K  P  F  I  D  I  G  V  E  Q  Y  T  R  K  L  P  G  C  E  E  F
1531 AAGGAAGCAATCAGAATCACCGGCCAGAAGCCATTCATCGACATCGGAGTGGAACAGTACACGCGTAAGCTGCCCGGCTGCGAGGAGTTC
 541 E  F  N  S  D  D  Y  W  R  C  Y  V  R  T  L  T  G  S  Y  Y  H  Y  V  G  T  C  K  M  G  P
1621 GAGTTCAACAGTGATGATTACTGGCGCTGCTATGTACGAACCCTAACCGGCTCCTATTATCATTATGTGGGAACGTGCAAAATGGGACCG
 571 K  S  D  P  S  A  V  V  D  A  R  L  R  V  Y  G  V  E  K  L  R  V  V  D  I  G  I  V  P  R
1711 AAATCAGATCCATCGGCTGTTGTCGATGCACGGCTAAGGGTTTATGGAGTGGAGAAATTACGAGTCGTCGATATTGGAATTGTACCGCGG
 601 P  P  S  A  H  T  A  A  M  A  Y  M  I  G  D  K  G  S  D  M  I  K  E  D  N  D  L  A  *
1801 CCGCCTTCAGCTCATACAGCCGCAATGGCGTACATGATCGGAGACAAAGGATCAGACATGATCAAGGAGGACAATGATCTTGCATGA
```

Amplicon size: 579 bp

ChtB4

```
  1 ACTAACTCTTACACTGAAAGGTCGT 5' FW
  1 *  L  R  M  *  L  S  S  N  A  K  I  C  T  N  L  C  V  I  S  N  K  C  H  L  G  R  S  S  V
  1 TGATTGAGAATGTGACTTTCCAGCAATGCTAAAATTTGCACTAATCTTTGTGTTATCAGCAACAAGTGCCATCTTGGGCGCTCCTCAGTT
 31 A  A  I  S  G  C  Q  R  N  R  V  A  K  I  Q  F  H  Q  Q  Q  R  S  R  L  P  V  Y  X  X  X
 91 GCAGCAATCTCAGGATGCCAACGGAATCGAGTTGCTAAGATACAATTCCACCAACAACAACGAAGCAGGCTACCAGTTTACNNNNNNNNN
 61 X  X  X  X  F  A  H  S  *  L  G  F  P  L  *  I  R  A  E  Q  Q  A  D  L  F  *  S  G  N  A
181 NNNNNNNNNNNATTCGCACACAGCTAACTTGGATTCCCCCTTTAGATACGAGCAGAGCAACAAGCAGATCTTTTCTGAAGTGGGAACGCC
 91 K  G  L  H  R  W  N  E  G  F  G  R  R  G  S  L  H  V  C  G  S  R  W  A  D  L  L  G  Q  L
271 AAAGGACTCCACAGATGGAACGAAGGTTTTGGCCGTAGAGGGAGCTTACACGTTTGTGGCTCCCGATGGGCAGACCTACTGGGTCAACTA
361                                                                 CCATAAGTTGGCCCTGTCCTACG 5' RV
121 S  C  R  R  E  R  I  P  T  E  D  G  N  W  Y  R  W  R  Y  S  T  G  T  G  C  P  G  P  E  L
361 TCGTGCCGACGAGAACGGATTCCTACCGAAGACGGGAACTGGTACCGTTGGAGGTATTCAACCGGGACAGGATGCCCCGGTCCGGAATTG
151 T  *  D  R  A  N  L  W
451 ACGTAGGACAGGGCCAACTTATGG
```

Amplicon size: 406 bp

Atro-1

```
   1 M  T  L  S  R  G  P  Y  S  E  L  D  K  M  S  L  F  Q  D  L  K  L  K  R  R  K  V  D  S  R
   1 ATGACACTGAGCCGTGGACCTTACAGCGAGCTCGATAAAATGAGCCTTTTCCAAGATTTGAAGCTGAAACGAAGAAAAGTTGACTCAAGA
  31 C  S  S  D  G  E  S  I  A  D  T  S  T  S  S  P  D  L  L  Q  P  L  S  P  K  M  C  D  Q  Q
  91 TGTAGCAGCGATGGAGAGTCTATAGCGGATACCAGCACTTCATCACCTGACCTTTTGCAGCCTTTGTCACCCAAGATGTGCGATCAGCAG
  61 Q  S  A  G  Q  L  E  L  D  D  P  T  E  L  E  D  G  S  P  A  P  T  A  A  A  N  H  H  H  H
 181 CAGTCGGCAGGCCAATTGGAGCTGGACGATCCCACCGAGCTCGAGGATGGCTCTCCAGCTCCAACGGCTGCTGCAAATCACCACCACCAC
  91 L  Q  H  H  P  T  H  H  H  P  H  H  H  H  H  H  P  H  S  N  N  M  D  N  N  N  T  L  T  S
 271 CTTCAACACCACCCAACTCACCACCACCCTCATCATCACCATCATCACCCCCATAGCAATAATATGGATAATAACAACACGTTAACAAGC
 121 N  N  N  N  N  D  C  I  T  S  P  K  L  E  H  D  G  S  M  M  K  D  D  D  G  D  S  E  R  A
 361 AACAATAACAACAACGACTGTATAACGTCACCAAAGTTAGAACATGATGGCAGCATGATGAAGGACGATGATGGCGACAGTGAAAGGGCG
 151 S  P  D  S  P  G  L  L  I  D  E  R  P  P  S  R  L  K  P  A  D  P  G  G  C  T  P  G  A  L
 451 TCACCAGATTCGCCTGGTTTGCTAATCGACGAACGTCCACCTTCGAGACTGAAACCTGCGGATCCTGGTGGTTGTACGCCGGGTGCACTT
 181 P  N  S  S  N  C  G  G  G  A  S  V  I  R  S  V  A  D  E  R  P  R  S  S  S  P  P  N  S  H
 541 CCTAATTCCTCCAATTGCGGTGGGGGCGCTAGTGTGATACGTTCGGTGGCGGATGAGCGTCCCCGGTCTTCAAGTCCGCCAAACAGCCAT
 211 N  L  A  L  M  N  S  I  Q  L  L  Q  Q  H  Y  S  A  S  G  N  A  G  S  S  S  S  S  S  S  I
 631 AATTTAGCGCTAATGAATTCCATTCAACTCCTGCAACAACACTACAGTGCAAGCGGTAACGCTGGTAGCAGTAGTAGTAGCAGCTCTATT
 241 G  A  A  H  S  V  P  G  H  Q  Q  Q  Q  H  V  T  V  L  V  T  P  S  R  M  K  P  D  H  P  T
 721 GGTGCTGCCCATAGTGTGCCAGGTCATCAGCAACAACAGCACGTGACCGTACTTGTCACCCCATCTCGAATGAAACCAGATCATCCGACG
 271 T  L  V  T  L  G  M  R  K  Q  A  P  P  S  I  N  L  A  N  T  S  N  N  T  N  S  S  S  T  N
 811 ACGTTGGTTACGCTAGGGATGCGAAAACAGGCGCCACCGTCAATTAATCTAGCAAATACCAGCAACAACACCAACAGCAGTAGCACTAAT
 301 S  S  N  N  C  N  G  S  N  S  N  V  G  S  N  K  N  E  E  P  N  R  L  T  S  T  P  Q  S  S
 901 AGTAGTAATAATTGTAACGGTAGTAATAGCAACGTTGGTAGTAATAAGAACGAAGAGCCTAATAGACTTACATCGACACCGCAGAGTTCA
 331 T  S  S  S  P  S  S  S  S  S  S  S  N  S  S  S  N  S  S  S  T  C  G  V  S  P  N  Q  A  N
 991 ACGTCGTCATCACCTTCATCTTCGTCGTCATCTTCGAACTCATCATCGAATTCGTCATCAACATGTGGTGTATCGCCAAATCAAGCAAAC
 361 Q  Q  P  V  H  L  Q  M  F  Q  Q  P  P  I  G  G  M  S  K  F  S  S  S  Q  S  L  S  N  N  S
1081 CAACAACCAGTCCATCTTCAGATGTTTCAACAGCCTCCTATCGGTGGCATGTCTAAGTTCTCATCTAGTCAATCTTTGTCAAACAATTCA
 391 N  I  Q  Q  Q  Q  Q  H  L  A  Q  Q  Q  M  L  Q  A  M  Q  Q  Q  I  R  I  K  K  E  R  M  P
1171 AATATCCAACAGCAGCAACAGCATCTCGCCCAACAACAAATGCTGCAAGCAATGCAACAGCAAATTCGGATAAAAAAGGAACGCATGCCG
 421 Q  Y  T  I  V  N  Q  N  T  N  M  S  L  M  H  G  N  Q  H  R  Q  Q  N  I  H  M  I  P  N  P
1261 CAATACACGATAGTTAACCAAAATACCAACATGTCATTAATGCATGGCAACCAACATCGCCAGCAGAACATTCACATGATTCCTAACCCA
 451 Q  Q  Q  L  Y  V  K  R  E  R  P  L  P  G  Q  S  H  T  V  V  N  M  P  P  V  S  M  A  G  P
1351 CAACAACAGCTGTATGTGAAACGTGAACGACCATTACCTGGTCAGAGTCACACAGTTGTGAACATGCCGCCTGTTTCAATGGCTGGTCCC
 481 A  N  H  Q  R  Q  P  P  S  S  P  V  Q  V  C  N  S  T  S  P  M  Q  P  P  M  S  P  S  Q  Q
1441 GCTAATCACCAAAGGCAACCTCCGAGTAGTCCTGTCCAAGTCTGCAATAGTACATCGCCAATGCAGCCACCAATGTCACCAAGTCAACAG
 511 H  Q  N  R  Q  Q  S  P  V  H  M  G  S  N  N  M  G  Q  S  P  H  I  I  I  K  R  E  R  Q  A
1531 CATCAAAACCGACAGCAAAGCCCTGTTCATATGGGGTCCAACAATATGGGCCAATCACCACATATTATCATCAAACGAGAACGGCAAGCA
 541 A  S  Q  S  M  T  N  A  M  Q  L  S  P  N  N  Q  R  L  P  S  P  I  Q  V  S  P  T  T  S  P
1621 GCTAGTCAAAGTATGACTAATGCTATGCAACTATCACCAAACAACCAGCGGTTACCAAGTCCTATTCAAGTCAGTCCTACCACGTCTCCA
 571 L  N  P  A  M  Q  I  R  H  P  M  R  D  A  A  I  L  F  R  V  K  N  E  A  Q  V  P  N  L  M
1711 CTAAATCCCGCCATGCAGATACGACACCCCATGCGGGATGCTGCCATATTATTTCGCGTAAAAAATGAAGCACAAGTACCAAATCTCATG
 601 Q  Q  Q  P  P  G  M  A  H  R  I  T  V  W  P  G  G  Q  Q  R  I  N  G  V  K  P  E  V  I  G
1801 CAACAGCAACCACCTGGAATGGCTCACCGGATAACCGTTTGGCCCGGTGGACAGCAGCGTATCAATGGCGTCAAACCGGAAGTGATCGGT
 631 G  P  L  P  P  L  R  N  Q  M  S  P  Q  Q  S  P  Q  H  H  Q  S  P  S  P  I  A  S  Q  T  P
1891 GGCCCGTTACCGCCGTTGCGAAACCAAATGTCCCCCCAACAGTCGCCGCAGCACCATCAAAGTCCTAGCCCTATCGCCTCGCAAACGCCA
 661 P  R  N  T  P  T  V  I  M  G  E  S  C  G  V  R  T  M  V  W  S  Y  D  S  P  P  Q  V  Q  T
1981 CCACGGAACACCCCGACAGTCATCATGGGCGAGTCGTGCGGTGTACGGACTATGGTCTGGAGCTACGACTCTCCACCGCAAGTACAAACC
 691 T  T  P  P  I  A  P  S  S  A  T  N  V  T  A  S  Q  G  P  L  G  P  G  S  S  L  G  M  G  T
2071 ACTACGCCACCAATTGCGCCATCTTCGGCGACAAACGTGACAGCATCGCAGGGTCCGCTAGGTCCTGGATCTTCGCTCGGGATGGGCACC
 721 I  P  P  S  P  G  N  H  H  H  Q  N  S  N  S  S  S  S  S  S  S  T  N  N  N  E  E  A  A  H
2161 ATTCCTCCCTCGCCGGGTAACCACCATCACCAAAACAGCAACAGCAGCAGTAGCAGCAGTAGTACCAACAACAACGAGGAGGCGGCGCAT
 751 L  L  L  S  L  G  Q  T  V  R  Q  N  E  A  Q  P  W  T  P  N  R  S  G  L  P  L  N  M  E  R
2251 CTACTTCTAAGCTTAGGACAAACCGTAAGGCAAAACGAGGCTCAACCATGGACTCCCAATCGATCCGGACTGCCCCTGAATATGGAACGG
 781 L  W  A  G  D  Y  T  Q  F  P  T  G  H  Q  M  H  A  L  N  L  T  S  Q  Q  P  W  N  M  P  P
2341 TTGTGGGCGGGAGATTATACCCAGTTCCCCACGGGACACCAAATGCATGCTCTCAATCTCACCTCGCAGCAACCGTGGAATATGCCACCG
 811 G  P  K  K  E  L  L  E  D  M  P  P  D  E  D  E  Q  P  L  V  C  M  I  C  E  D  K  A  T  G
2431 GGTCCAAAAAAGGAATTGCTTGAGGACATGCCTCCTGACGAGGATGAACAACCGTTGGTGTGCATGATCTGCGAAGACAAAGCCACCGGA
 841 L  H  Y  G  I  I  T  C  E  G  C  K  G  F  F  K  R  T  V  Q  N  R  R  V  Y  T  C  V  A  D
2521 TTGCACTACGGCATCATCACCTGCGAAGGCTGCAAAGGATTCTTCAAGCGAACCGTGCAAAACCGAAGGGTTTACACGTGCGTCGCGGAT
2611                                                                     GCAAGGAATGGTGCTTCAAGCT
 871 G  T  C  E  I  T  K  A  Q  R  N  R  C  Q  Y  C  R  F  K  K  C  I  E  Q  G  M  V  L  Q  A
2611 GGTACCTGTGAAATAACTAAGGCCCAGCGCAACCGCTGCCAGTATTGTAGATTTAAGAAGTGTATCGAGCAAGGAATGGTGCTTCAAGCT
2701 G 3' FW
 901 V  R  E  D  R  M  P  G  G  R  N  S  G  A  V  Y  N  L  Y  K  V  K  Y  K  K  H  K  K  N  N
2701 GTTCGAGAAGATCGTATGCCAGGAGGACGAAACAGTGGCGCGGTTTACAACCTGTACAAAGTAAAATATAAGAAACACAAGAAAAACAAC
 931 Q  K  G  N  N  H  Q  K  L  G  M  P  G  D  N  P  K  S  M  F  L  T  S  S  T  T  P  P  I  K
2791 CAGAAAGGTAACAACCACCAAAAACTCGGCATGCCCGGTGACAATCCAAAGTCGATGTTCTTGACGTCATCGACGACGCCGCCGATCAAG
 961 S  E  S  I  S  L  P  S  H  L  V  N  G  T  I  L  K  T  A  L  T  N  P  S  E  I  V  H  L  R
2881 TCGGAAAGTATTAGTCTGCCATCGCACCTGGTCAATGGAACGATACTGAAAACTGCCCTGACCAATCCAAGCGAAATCGTGCATTTACGC
 991 H  R  L  D  N  A  V  S  S  S  K  D  R  S  I  S  Y  E  Q  A  L  N  M  I  H  T  L  I  D  C
2971 CATAGACTGGACAACGCCGTCAGCTCGTCCAAGGATCGGTCGATCTCGTACGAGCAAGCCTTGAACATGATACATACCCTTATAGACTGT
1021 D  A  M  E  D  I  A  T  L  P  H  F  S  E  F  L  A  D  K  S  E  I  G  D  K  L  C  N  I  G
3061 GATGCTATGGAGGACATAGCGACCCTACCGCATTTAGTGAATTCCTCGCCGACAAGTCGGAAATTGGCGATAAGCTCTGTAATATAGGT
```

```
3151                           CTGAGCGTTCAACGGGAAGATGT 5' RV
1051 D  S  I  V  H  K  L  V  S  W  T  R  K  L  P  F  Y  M  D  I  P  V  E  I  H  T  K  L  L  T
3151 GATTCGATTGTACATAAGCTCGTCTCGTGGACTCGCAAGTTGCCCTTCTACATGGACATCCCGGTAGAGATCCACACAAAGCTACTGACA
1081 D  K  W  H  E  I  L  V  L  T  T  A  A  Y  Q  A  L  H  G  S  K  S  I  G  S  Q  L  Q  S  S
3241 GACAAATGGCACGAGATACTGGTGCTGACGACCGCCGCCTATCAGGCCTTACACGGCAGCAAAAGCATAGGTAGCCAGCTCCAATCCTCG
1111 T  G  G  N  G  P  G  G  G  T  V  L  A  P  D  K  Q  D  S  E  F  V  E  E  I  T  A  H  L  H
3331 ACCGGCGGGAACGGTCCGGGAGGTGGAACCGTTCTCGCACCTGACAAACAAGACTCCGAGTTTGTCGAAGAGATCACAGCCCACCTGCAT
1141 T  L  Q  S  C  L  T  T  L  M  G  H  P  I  S  I  E  Q  L  K  I  D  V  G  Q  M  V  E  K  M
3421 ACGTTACAATCCTGCCTAACCACCCTAATGGGTCATCCGATTTCGATCGAACAACTGAAAATTGACGTCGGTCAAATGGTTGAGAAGATG
1171 T  Q  I  T  I  M  F  R  R  S  K  L  K  M  E  E  Y  V  C  L  K  V  Y  I  L  L  N  K  E  V
3511 ACCCAAATTACGATAATGTTCCGACGGAGTAAACTCAAGATGGAAGAGTACGTCTGTCTCAAGGTTTACATTCTGTTGAACAAAGAAGTC
1201 E  L  E  S  I  Q  E  R  Y  V  Q  V  L  R  T  Y  L  Q  H  T  V  P  H  S  P  N  R  L  A  D
3601 GAATTGGAGAGCATACAGGAACGGTACGTTCAAGTACTTAGGACTTATCTACAGCACACCGTACCACACAGTCCAAATCGACTGGCGGAC
1231 L  L  S  H  I  P  E  I  Q  T  A  A  S  L  L  L  E  S  K  M  F  Y  V  P  F  V  L  N  S  A
3691 CTGTTATCCCATATACCTGAGATCCAAACCGCTGCCAGCTTACTACTGGAGAGTAAAATGTTCTACGTACCGTTCGTTCTGAACTCTGCC
1261 N  I  R  *
3781 AATATTAGGTAG
```

Amplicon size: 524 bp

DHR4

```
   1 M  M  L  K  L  L  I  A  A  C  L  V  S  H  G  L  G  R  P  D  V  S  H  I  L  K  Q  E  N  Q
   1 ATGATGCTGAAACTGCTGATAGCGGCGTGCTTGGTATCGCACGGTCTTGGACGACCCGATGTTTCACACATTTTGAAGCAGGAAAACCAA
  31 R  Q  N  D  R  A  F  H  P  L  D  Y  L  S  R  Q  L  G  K  G  G  E  F  E  A  R  A  A  T  A
  91 CGCCAGAACGACAGGGCCTTCCATCCGCTGGATTACCTCAGTCGGCAGTTGGGCAAAGGTGGCGAGTTTGAAGCCCGTGCTGCCACCGCG
  61 S  E  Y  N  V  F  D  Y  L  T  A  F  N  E  T  E  I  N  G  L  E  G  R  S  S  P  P  I  G  G
 181 AGTGAGTACAATGTGTTCGATTATTTAACTGCTTTTAATGAAACGGAAATTAACGGTCTTGAAGGCAGATCATCACCTCCCATTGGCGGT
  91 R  R  A  G  N  R  A  F  R  N  V  V  K  P  S  P  F  K  K  N  T  A  E  F  G  Y  T  Y  E  K
 271 AGACGGGCTGGTAACAGAGCTTTTAGGAATGTCGTTAAACCCTCTCCATTCAAGAAGAACACTGCTGAATTTGGCTACACCTATGAGAAA
 121 P  S  D  P  F  D  F  P  I  N  P  D  A  N  L  I  K  P  V  S  T  Q  A  P  E  Y  L  P  P  V
 361 CCATCCGATCCATTTGACTTCCCGATCAATCCAGATGCTAACTTGATTAAGCCAGTATCAACTCAAGCTCCAGAATATCTGCCTCCGGTG
 151 T  Q  S  P  P  T  P  A  D  V  Q  S  S  V  S  G  I  P  S  S  P  S  Y  P  G  L  V  P  G  S
 451 ACACAAAGTCCTCCAACACCTGCAGATGTACAATCTTCGGTTTCCGGTATACCTTCTTCGCCATCGTACCCTGGATTGGTACCTGGTTCT
 181 T  D  T  E  R  P  Q  R  P  I  E  S  T  S  T  Q  G  S  V  A  S  I  G  T  S  T  S  S  P  T
 541 ACAGATACCGAAAGGCCTCAGAGACCAATTGAATCTACATCTACGCAAGGATCAGTAGCAAGTATTGGAACTAGCACTTCCTCTCCTACA
 631                                             ATGTCACAATCCCCACAGAACGG 3' FW
 211 P  G  S  E  T  P  S  F  P  G  M  S  Q  S  P  Q  N  G  Y  S  P  P  G  P  T  S  S  P  V  D
 631 CCAGGATCGGAGACACCTAGTTTTCCTGGTATGTCACAATCCCCACAGAACGGTTACAGCCCACCAGGACCCACATCTAGTCCAGTAGAC
 241 L  T  T  G  Y  Q  Q  G  S  T  A  D  D  E  F  T  G  Y  P  P  A  S  S  P  E  P  G  L  S  S
 721 TTGACTACTGGATACCAACAAGGATCTACTGCCGATGATGAATTTACAGGATATCCTCCAGCATCTTCTCCTGAGCCCGGTCTTTCTTCA
 271 S  V  Y  P  P  E  S  R  P  S  I  I  P  G  S  P  V  S  T  T  F  P  Q  E  T  P  T  S  T  S
 811 AGTGTTTATCCTCCGGAATCAAGGCCTAGTATTATCCCAGGAAGTCCAGTATCAACGACTTTTCCACAGGAAACTCCTACGAGTACTTCC
 301 Q  Q  P  E  S  Q  V  T  T  E  S  G  F  T  G  Y  P  P  Y  K  P  S  S  E  S  T  P  R  P  S
 901 CAACAGCCTGAATCTCAAGTAACCACCGAAAGTGGATTTACTGGTTATCCTCCGTATAAGCCTAGTTCAGAATCTACACCAAGACCTTCT
 331 P  S  S  T  D  Y  P  Q  T  S  S  S  L  P  G  Q  Q  P  S  T  T  P  G  F  T  G  Y  P  E  S
 991 CCGTCATCAACCGATTATCCACAAACATCCTCTAGCTTACCAGGTCAACAACCATCAACCACACCGGGATTCACTGGGTATCCTGAAAGT
1081                                                                    CCGGTTCGCTTGTTGGTCTTTAG
 361 F  S  S  T  V  S  P  D  Q  D  S  G  T  T  T  S  P  T  G  Y  P  Q  G  P  S  E  Q  P  E  I
1081 TTCAGCTCAACAGTATCGCCTGATCAAGATTCTGGAACTACTACCTCTCCAACTGGTTATCCACAAGGGCCAAGCGAACAACCAGAAATC
1171  5' RV
 391 G  F  T  G  Y  P  Q  G  P  S  S  T  V  S  P  G  E  T  S  T  A  L  T  E  S  E  T  T  T  E
1171 GGATTTACTGGATATCCTCAAGGACCAAGTTCAACAGTTTCACCTGGAGAAACTAGTACAGCTTTAACGGAATCTGAAACGACTACAGAA
 421 K  E  I  G  G  Y  P  Q  G  P  F  S  P  N  A  T  T  E  S  P  I  T  D  S  T  P  A  L  L  T
1261 AAAGAAATTGGTGGATATCCACAGGGTCCATTCTCACCAAACGCTACAACCGAAAGTCCCATTACCGATTCCACCCCGGCTTTGCTCACA
 451 T  E  G  T  Q  P  G  K  P  V  L  P  P  N  L  N  E  V  L  G  E  P  F  D  I  N  D  L  A  T
1351 ACCGAGGGTACTCAACCAGGTAAACCAGTGCTTCCACCCAACCTAAATGAGGTGCTTGGAGAGCCCTTCGACATCAACGATCTTGCCACT
 481 T  T  Q  P  T  T  T  T  T  P  K  T  A  D  G  S  F  I  P  P  K  P  R  P  E  L  V  V  P  N
1441 ACTACGCAGCCAACAACCACCACTACTCCAAAAACGGCCGATGGATCCTTCATTCCTCCAAAACCTCGCCCGGAGTTGGTTGTTCCGAAT
 511 A  P  S  A  S  P  I  P  D  Q  T  E  R  P  A  T  Q  P  T  Q  L  T  T  Q  A  A  L  P  G  Q
1531 GCACCGAGTGCCTCGCCGATCCCTGATCAAACTGAAAGACCCGCAACTCAACCAACACAATTGACAACACAGGCAGCCTTGCCTGGACAA
 541 P  S  S  T  S  A  P  E  Y  L  P  P  D  S  D  S  P  Q  S  P  Q  T  G  P  S  T  T  G  F  G
1621 CCATCTTCCACTTCTGCTCCAGAATACCTGCCACCGGATAGTGATAGTCCTCAGAGTCCTCAAACAGGTCCTTCAACTACTGGTTTTGGA
 571 T  S  E  S  V  T  T  E  A  N  Y  P  S  S  S  L  E  T  T  T  F  A  G  Y  P  S  F  D  R  D
1711 ACCAGCGAATCTGTTACTACAGAAGCCAACTATCCAAGTTCTAGTCTAGAAACCACAACATTTGCCGGCTATCCGAGTTTCGATCGCGAT
 601 T  T  T  S  T  R  Y  P  V  V  G  Q  D  T  S  T  T  V  G  Y  A  S  Q  E  T  T  T  F  A  G
1801 ACAACGACTTCCACCCGCTATCCAGTTGTAGGTCAAGACACTTCAACTACTGTGGGTTATGCTAGTCAGGAAACCACAACCTTTGCGGGT
 631 Y  P  E  S  D  R  E  T  T  T  T  S  S  Y  L  G  P  N  Q  E  A  S  T  S  S  S  Y  P  S  S
1891 TATCCAGAATCTGATCGTGAAACCACGACTACGTCAAGTTATTTGGGCCCAAATCAGGAAGCTTCCACATCTTCTAGCTACCCAAGTTCC
 661 D  R  D  T  T  T  P  V  S  Y  P  K  P  V  Q  E  A  S  P  D  L  G  Y  P  V  S  N  G  N  R
1981 GATCGTGATACCACAACTCCTGTTAGCTACCCAAAACCAGTTCAAGAAGCTTCTCCTGATTTAGGATACCCTGTTTCCAATGGTAATCGT
 691 T  T  T  S  S  Y  P  S  A  G  P  E  T  S  T  A  A  G  Y  P  S  P  D  S  A  T  Q  V  V  Q
2071 ACGACTACGTCAAGCTATCCTAGTGCAGGACCAGAAACTTCAACAGCTGCTGGATATCCTAGTCCGGATAGTGCAACGCAGGTTGTTCAA
 721 E  T  T  T  A  T  D  F  S  S  A  S  Q  E  T  T  T  S  S  R  Y  P  S  E  E  T  I  T  T  S
2161 GAAACCACAACAGCCACAGATTTCTCAAGTGCGAGTCAGGAAACCACGACCTCTTCTAGGTACCCGAGTGAAGAGACCATAACTACTTCT
 751 R  P  E  T  T  T  F  G  G  Y  P  N  A  Q  G  S  T  T  G  A  A  E  Y  T  T  V  S  T  I
2251 AGACCAGAAACAACCACCTTTGGTGGCTATCCAAATGCACAGGGATCCACAACCACTGGTGCTGCTGAATACACCACAGTATCTACGATA
 781 G  L  E  Y  S  T  G  Q  P  S  T  S  K  P  L  D  G  A  R  P  Y  P  M  G  P  G  S  D  Q  S
2341 GGTCTAGAATATTCTACGGGTCAGCCGTCAACTTCAAAACCACTTGATGGGGCTCGACCATACCCAATGGGTCCTGGCTCAGACCAATCT
 811 S  G  P  M  S  T  A  A  P  E  Y  L  P  P  E  D  D  N  S  T  S  Q  Y  Y  P  P  A  T  S  P
2431 TCCGGGCCAATGTCTACCGCAGCTCCTGAGTATCTTCCACCAGAAGATGATAACTCCACCAGTCAGTATTATCCACCTGCGACCAGCCCG
 841 A  V  E  F  T  S  S  V  P  E  Y  R  P  E  Q  P  V  R  P  S  I  P  S  D  S  Q  V  N  Q  A
2521 GCAGTAGAATTCACTTCAAGCGTTCCGGAGTACCGTCCAGAGCAGCCTGTTCGCCCCTCAATTCCTTCCGACAGTCAAGTTAATCAGGCT
 871 V  P  A  S  P  D  Y  T  E  P  P  T  D  E  Q  Q  P  E  Q  P  E  T  P  I  N  I  M  E  M  I
2611 GTCCCCGCAAGTCCTGATTATACCGAGCCACCAACAGATGAACAACAACCTGAACAACCGGAAACTCCCATCAACATAATGGAAATGATC
 901 N  A  I  N  G  L  V  P  G  N  S  Y  L  P  P  S  S  I  N  M  S  D  L  R  I  V  V  D  D  T
2701 AACGCAATCAATGGCCTTGTTCCTGGCAATAGCTATCTACCGCCATCTTCAATTAACATGTCTGATCTGCGAATCGTGGTTGATGACACG
 931 P  S  S  D  P  I  Q  T  P  P  S  S  P  S  G  P  A  S  E  A  E  A  D  G  S  E  V  Q  P  R
2791 CCGTCTTCCGATCCAATCCAAACCCCTCCCAGCAGTCCCAGTGGGCCGGCATCGGAAGCGGAAGCCGACGGGTCTGAGGTGCAGCCTAGG
 961 V  A  L  A  E  S  T  S  K  G  Y  N  Y  Q  V  P  E  E  R  L  P  V  P  V  I  P  S  H  T  L
2881 GTGGCCTTGGCAGAATCTACCAGCAAAGGCTACAACTATCAAGTACCGGAGGAACGGTTACCTGTTCCAGTAATACCTTCGCATACGCTC
 991 D  D  E  G  Y  H  Y  K  I  P  K  V  P  F  L  T  *  V
2971 GACGACGAGGGCTACCACTACAAAATCCCAAAAGTTCCTTTCCTCACCTAGGTTTA
```

Amplicon size: 510 bp

GALNT6

```
  1 GGCTGTGAATGGTCTGCAACGAA 5' FW
  1 P  T  L  T  R  R  C  L  C  G  P  *  Q  Q  *  R  Y  S  Y  *  L  A  K  Q  *  E  V  S  L  K
  1 CCGACACTTACCAGACGTTGCTTATGTGGACCTTAACAACAATAGCGCTATTCCTACTGATTAGCCAAGCAATAGGAAGTGAGCTTAAAG
 31 G  S  *  R  T  I  A  P  A  T  T  *  T  *  R  A  I  H  *  W  T  S  H  R  V  F  H  Q  L  G
 91 GGCTCATAGCGTACGATTGCACCAGCAACAACGTAAACATAACGAGCTATTCACTGATGGACGTCGCATCGTGTATTCCACCAACTAGGA
 61 I  S  R  L  R  K  S  A  Y  K  S  Y  K  E  A  Q  K  H  S  S  R  S  T  S  A  R  *  *  *  N
181 ATCTCACGACTACGGAAGTCCGCATACAAGTCTTACAAAGAAGCCCAAAAACATTCGTCAAGGTCCACCAGTGCAAGGTAATAATAAAAC
271                  CATGTTGAGAGCTAAGCGACGGT 5' RV
 91 D  Q  S  D  T  A  Y  N  S  R  F  A  A
271 GATCAATCCGACACTGCGTACAACTCTCGATTCGCTGCCA
```

Amplicon size: 506 bp

ChtBD2

```
  1                               ACGGTGTTGGTGGTTTTCTCGAT 3' FW
  1 M  N  V  I  V  F  V  G  L  T  T  V  L  V  V  F  S  I  H  A  G  S  G  S  S  E  R  D  N  R
  1 ATGAACGTAATCGTGTTCGTCGGATTGACGACGGTGTTGGTGGTTTTCTCGATACACGCTGGAAGCGGTTCTTCAGAACGTGACAATCGA
 31 Q  G  L  D  W  C  S  S  F  P  D  E  E  A  R  D  E  C  A  D  S  E  G  T  F  W  M  Y  S  P
 91 CAAGGTCTGGATTGGTGCAGCTCATTCCCGGATGAAGAAGCACGCGACGAATGCGCGGATTCCGAGGGTACATTCTGGATGTATTCGCCG
 61 T  C  C  N  V  K  W  E  C  V  N  G  E  Y  N  Q  G  L  R  C  S  V  S  G  F  I  P  D  S  E
181 ACCTGCTGCAACGTGAAGTGGGAGTGTGTCAATGGAGAGTACAACCAGGGCTTAAGATGTAGTGTTTCTGGATTCATTCCGGATAGTGAA
 91 T  V  K  C  I  E  E  P  T  C  I  D  T  S  S  T  V  A  P  T  T  T  T  T  V  R  I  T  T  T
271 ACGGTGAAGTGCATTGAGGAGCCTACCTGTATTGATACTTCAAGTACTGTAGCTCCTACGACAACTACCACAGTGCGGATAACAACTACC
121 T  L  E  P  T  T  V  T  T  I  T  V  G  E  T  Q  F  C  D  T  F  P  L  D  E  P  P  I  Y  C
361 ACACTCGAACCGACTACAGTTACTACAATTACAGTTGGAGAGACGCAATTCTGTGATACATTTCCCTTGGACGAGCCTCCTATCTACTGC
451                                                                AACCTACCACGGATGTTGGTTCC 5'
151 A  E  T  S  G  S  F  W  M  S  S  P  S  C  C  N  V  K  F  E  C  L  D  G  A  Y  N  Q  G  F
451 GCTGAAACAAGCGGATCATTCTGGATGTCGTCGCCCAGCTGTTGTAACGTCAAATTCGAGTGTTTGGATGGTGCCTACAACCAAGGCTTC
541 RV
181 R  C  S  S  P  G  F  I  P  D  L  Q  R  L  K  C  I  E  D  S  T  C  V  R  K  E  E  T  P  S
541 CGTTGTAGCAGTCCTGGATTCATACCCGACCTCCAGCGATTGAAATGCATTGAAGACAGTACTTGCGTACGGAAGGAAGAAACTCCGAGT
211 E  D  T  D  N  S  S  P  D  I  I  C  S  N  V  S  T  E  Y  L  P  H  P  S  D  C  S  K  Y  F
631 GAAGATACAGACAACTCGTCTCCGGATATCATCTGCTCCAACGTCAGTACCGAGTATCTGCCCCATCCGAGTGACTGTTCAAAATATTTC
241 R  C  H  N  G  I  V  Q  Q  L  E  C  M  D  G  S  I  F  S  Y  Q  F  Q  Q  C  L  P  G  D  K
721 CGCTGCCACAATGGGATAGTGCAGCAGCTGGAGTGCATGGACGGATCAATCTTCAGCTACCAGTTCCAACAGTGTCTACCAGGAGATAAA
271 D  T  C  E  M  L  G  V  *
811 GACACTTGTGAAATGCTCGGCGTATGA
```

Amplicon size: 559 bp

KSPI

```
  1 CGCTTTTCGTTACGTGTGACTAA 5' FW
  1 A  K  S  N  A  H  *  L  C  F  R  S  R  F  C  W  D  *  F  *  W  L  A  L  *  L  R  K  L  V
  1 GCGAAAAGCAATGCACACTGATTATGCTTCCGAAGTCGGTTTTGTTGGGATTAATTCTAGTGGCTGGCTCTATAGCTCAGGAAACTGGTT
 31 A  S  A  V  T  R  *  F  P  F  A  A  R  M  E  *  P  T  R  I  P  A  T  W  S  A  C  R  S  E
 91 GCTTCTGCGGTGACGAGGTGATTCCCGTTTGCGGCTCGGATGGAGTGACCTACGAGAATACCTGCCACCTGGAGTGCTTGCAGAAGCGAA
181                                                 AGCCCTTTTCGACAAGTCACAGA 5' RV
 61 P  E  F  G  W  S  R  L  G  T  V  G  G  I  R  I  S  G  K  A  V  Q  C
181 CCGGAGTTCGGTTGGTCAAGGTTGGGAACTGTCGGGGGGATCCGGATCTCGGGAAAAGCTGTTCAGTGTCT
```

Amplicon size: 502 bp

Na^+/Ca^{2+}

```
   1 M  E  I  L  G  T  S  A  S  I  W  L  V  V  F  V  C  S  V  N  S  I  Y  V  P  S  L  F  D  F
   1 ATGGAGATTCTTGGGACTAGTGCTAGCATCTGGCTTGTGGTGTTTGTTTGCAGCGTGAATTCGATCTACGTTCCATCGCTGTTTGATTTC
  31 I  N  H  M  P  M  S  N  D  S  S  L  N  E  T  T  L  L  L  D  W  D  S  R  A  S  I  E  F  L
  91 ATCAATCACATGCCGATGTCGAATGACTCCTCACTCAACGAGACCACACTCCTCCTGGACTGGGACAGCCGGGCCAGCATAGAGTTTCTC
  61 P  G  A  N  E  S  L  S  P  V  V  C  V  L  T  T  S  M  D  D  L  P  P  D  L  F  S  K  T  E
 181 CCGGGAGCCAATGAATCTCTTTCCCCAGTGGTTTGTGTCCTTACAACATCGATGGATGATCTGCCACCGGATCTCTTCTCGAAAACCGAA
  91 R  L  N  G  A  I  I  L  H  F  L  A  A  I  Y  F  F  T  I  L  A  Y  V  C  S  E  Y  F  L  P
 271 CGGCTTAACGGAGCGATCATACTTCACTTCTTAGCTGCCATCTACTTTTTTACGATATTAGCCTACGTATGTAGCGAGTACTTCTTGCCA
 121 S  V  E  Y  I  C  E  D  L  H  L  S  E  D  V  A  A  A  T  F  M  A  T  A  T  S  M  P  E  F
 361 TCGGTGGAGTACATCTGTGAAGATCTGCACCTGTCTGAGGATGTAGCTGCAGCAACTTTTATGGCGACGGCAACATCTATGCCGGAATTC
 151 F  T  N  T  I  S  T  L  V  V  D  S  D  M  G  L  G  T  I  M  G  S  M  L  F  N  T  L  G  V
 451 TTCACAAACACCATCAGTACCTTGGTGGTCGATTCAGACATGGGCTTGGGGACCATCATGGGCTCCATGCTGTTCAACACTTTGGGAGTT
 541                                CACGTGAAACTTGATTGGTGGCC 3' FW
 181 A  A  L  V  G  L  L  T  K  S  H  V  K  L  D  W  W  P  L  T  R  D  S  I  I  V  I  I  S  T
 541 GCCGCATTAGTTGGTTTGTTAACCAAATCGCACGTGAAACTTGATTGGTGGCCGTTAACACGCGACTCGATCATCGTTATCATCAGTACA
 211 S  S  L  V  S  C  L  W  D  E  R  V  Y  W  Y  E  S  L  V  F  V  V  L  Y  V  L  Y  F  L  V
 631 AGCTCACTGGTTTCTTGCCTTTGGGATGAACGAGTCTACTGGTATGAATCGCTCGTTTTCGTCGTTCTCTACGTACTATATTTCTTAGTC
 241 M  F  Q  N  D  R  M  K  R  I  A  V  S  L  I  E  D  R  W  N  L  C  R  R  L  E  K  K  S  S
 721 ATGTTCCAAAACGATCGCATGAAGCGAATAGCAGTTTCCTTGATCGAGGATCGTTGGAATCTCTGCCGAAGGCTGGAGAAGAAATCTTCC
 271 D  E  P  Q  S  E  Y  K  N  H  R  K  Y  S  I  A  V  L  G  E  A  I  N  T  N  L  R  I  P  T
 811 GATGAACCCCAATCGGAATATAAAAACCATCGAAAGTACTCGATAGCCGTGCTGGGAGAGGCCATCAATACCAACTTACGTATCCCAACC
 301 S  D  K  E  A  P  R  I  S  Y  D  S  S  G  E  S  D  Q  S  D  D  S  R  M  H  L  L  K  I  S
 901 AGTGATAAGGAAGCTCCTCGTATCTCGTACGATTCCAGTGGCGAATCGGATCAATCCGACGATAGCAGAATGCATCTGCTGAAAATATCG
 331 K  D  S  W  L  G  V  F  W  W  F  Y  T  W  P  F  R  V  I  V  N  V  T  I  P  D  P  R  K  H
 991 AAAGACTCGTGGCTGGGAGTTTTCTGGTGGTTTTACACCTGGCCTTTCCGGGTGATAGTCAACGTAACGATTCCAGATCCCCGGAAGCAT
1081                            ATACACGTATCGCACCTAGCCAC 5' RV
 361 R  K  L  Y  P  L  T  F  F  M  C  I  A  W  I  G  G  T  A  Y  M  V  F  W  M  M  T  I  I  G
1081 CGTAAATTATATCCGTTGACATTCTTTATGTGCATAGCGTGGATCGGTGGTACAGCTTACATGGTGTTCTGGATGATGACGATCATTGGA
 391 S  T  F  D  V  P  E  T  V  M  G  L  T  F  L  A  F  G  G  C  M  P  E  A  V  S  A  I  T  V
1171 AGTACGTTTGATGTACCGGAAACGGTTATGGGACTTACCTTCCTCGCGTTCGGCGGATGCATGCCCGAGGCAGTTTCGGCCATTACGGTG
 421 I  R  K  G  N  G  S  M  G  V  S  N  S  L  G  A  N  T  L  A  I  L  F  S  L  G  L  P  W  F
1261 ATAAGAAAAGGAAATGGCTCCATGGGCGTATCGAACTCGCTGGGTGCAAACACCCTAGCAATCCTGTTCTCATTAGGACTACCGTGGTTC
 451 I  R  N  M  M  E  G  G  A  T  T  G  A  Y  I  E  I  N  S  Y  G  M  Q  Y  S  V  L  A  L  F
1351 ATCCGGAATATGATGGAAGGAGGAGCCACCACTGGAGCTTACATCGAAATAAATTCCTATGGAATGCAGTATTCGGTGTTGGCTCTATTT
 481 L  A  V  G  I  L  Y  L  V  L  Y  L  S  K  F  T  L  R  K  L  V  G  L  A  L  L  I  A  Y  L
1441 CTAGCAGTAGGGATTCTCTATCTGGTACTTTATTTATCAAAGTTCACGCTACGAAAGTTGGTAGGATTAGCACTGTTGATTGCGTACTTG
 511 I  I  V  S  F  M  I  L  V  E  L  D  V  F  F  P  S  K  N  I  C  *  I
1531 ATCATTGTCTCTTTTATGATTCTAGTCGAGTTAGATGTATTTTTTCCGTCGAAAAATATTTGTTGAATATA
```

Amplicon size: 564 bp

Figure 1. DNA and amino acid sequences of the eight selected transcripts use in the RNAi studies described in Materials and Methods. Primers specific for each sequence are shown in red

Gld

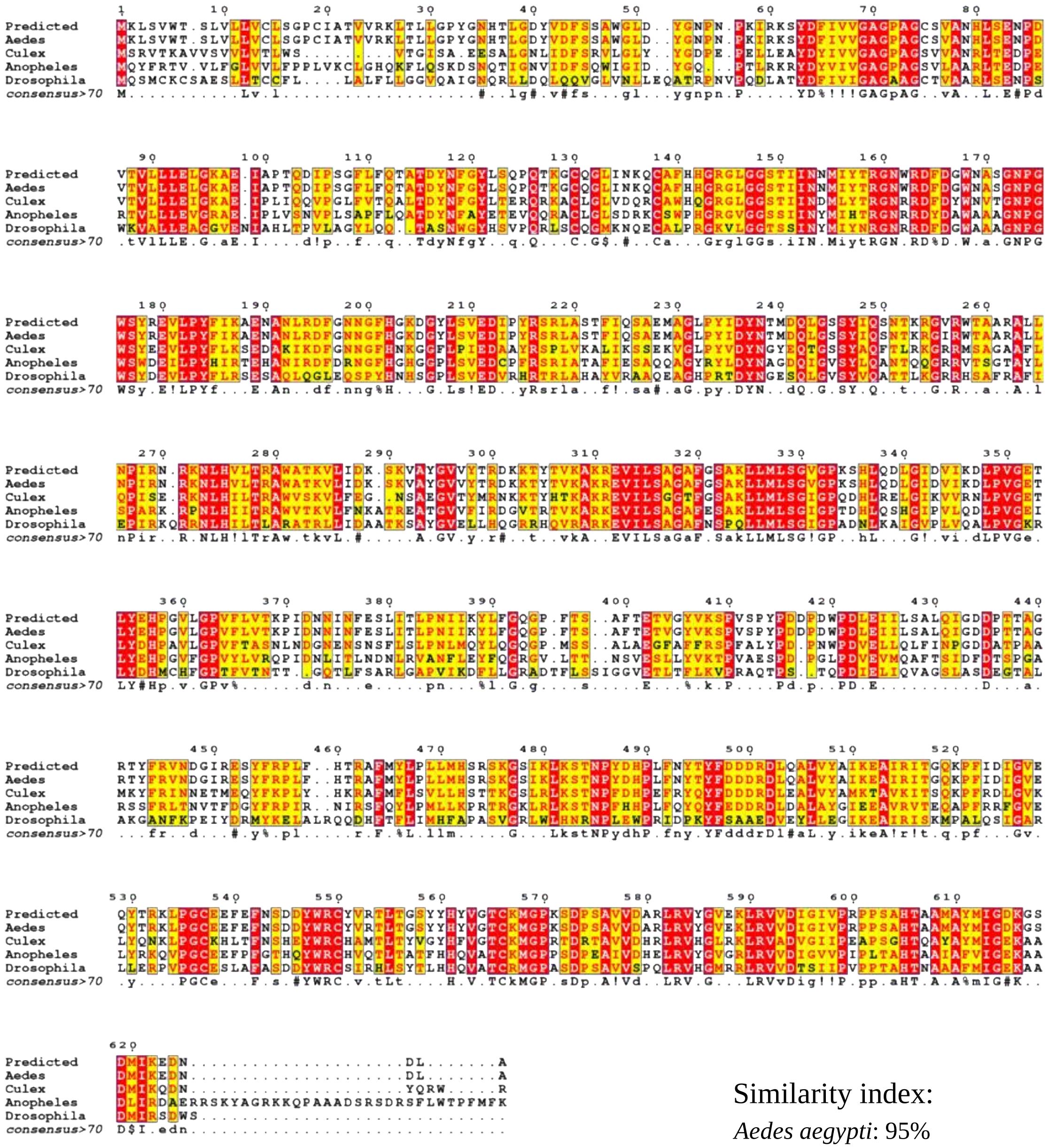

Similarity index:

Aedes aegypti: 95%
Culex quinquefasciatus: 95%
Anopheles darlingi: 94%
Drosophila obscura: 90%

ChtB4

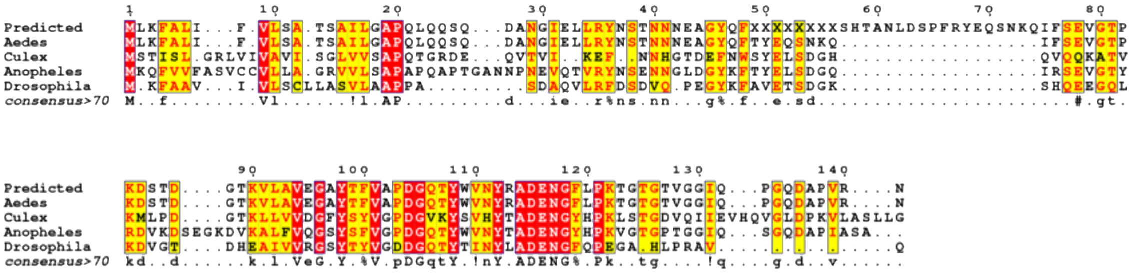

Similarity index:

Aedes aegypti: 85%
Culex quinquefasciatus: 75%
Anopheles albimanus: 81%
Drosophila serrata: 78%

Atro-1

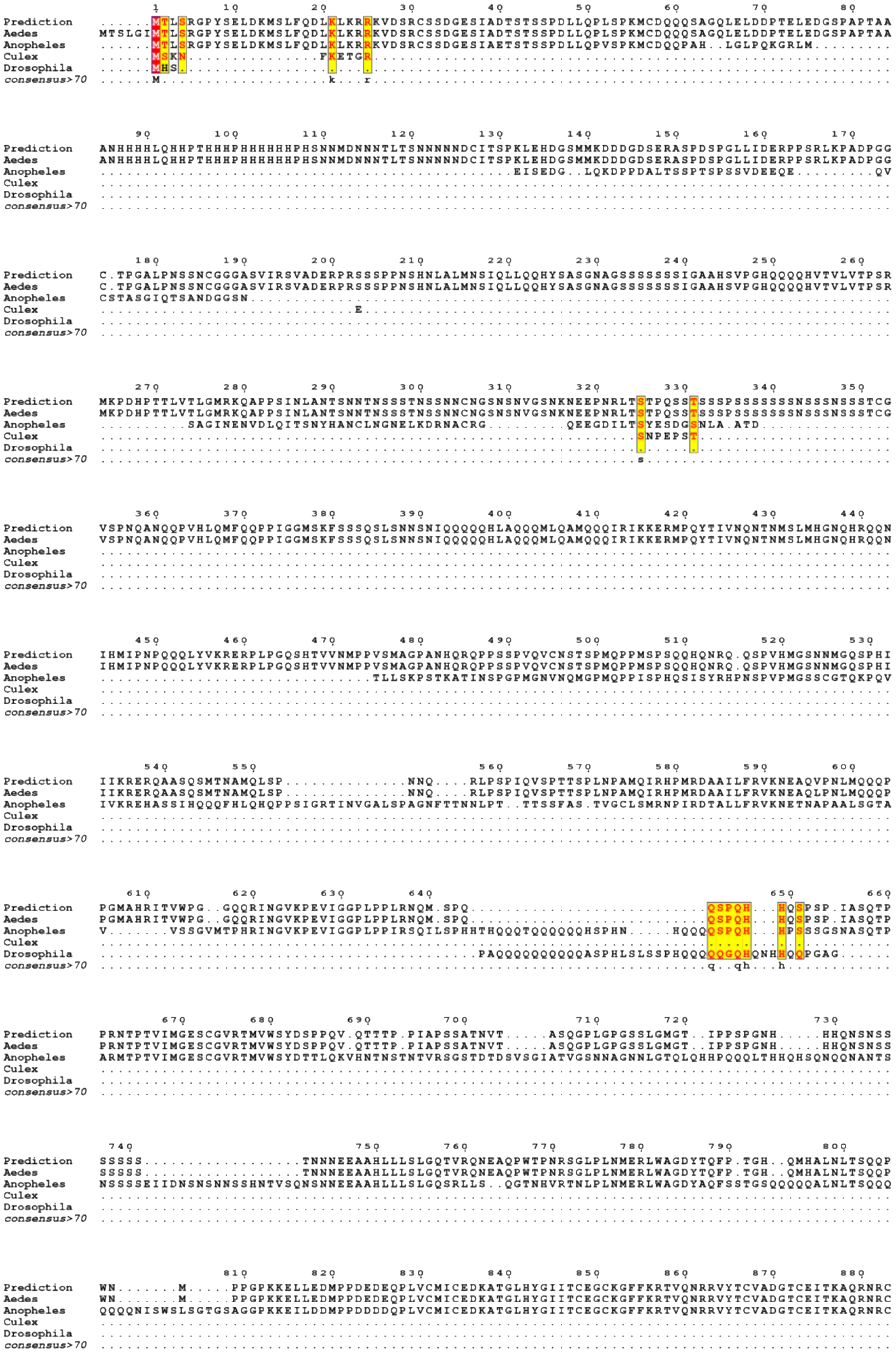

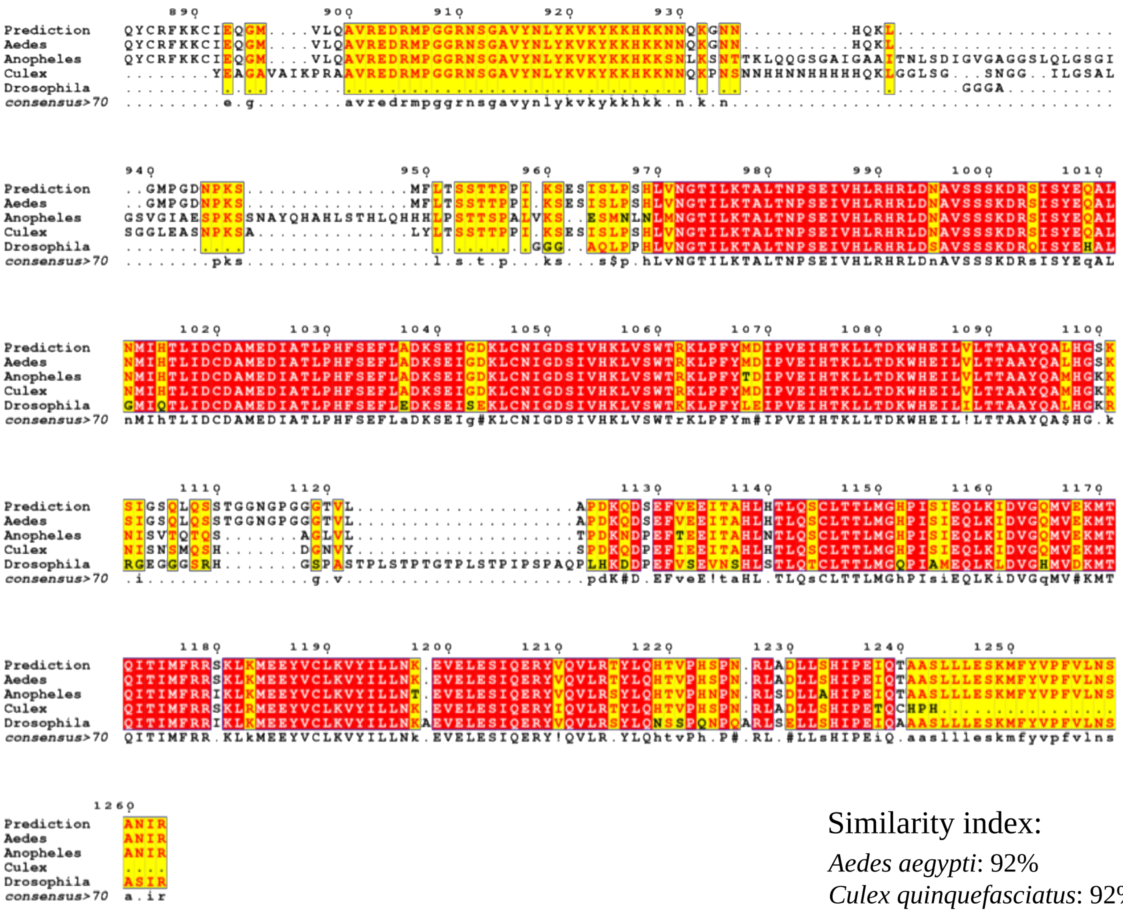

Similarity index:

Aedes aegypti: 92%
Culex quinquefasciatus: 92%
Anopheles gambiae: 84%
Drosophila sechellia: 92%

DHR4

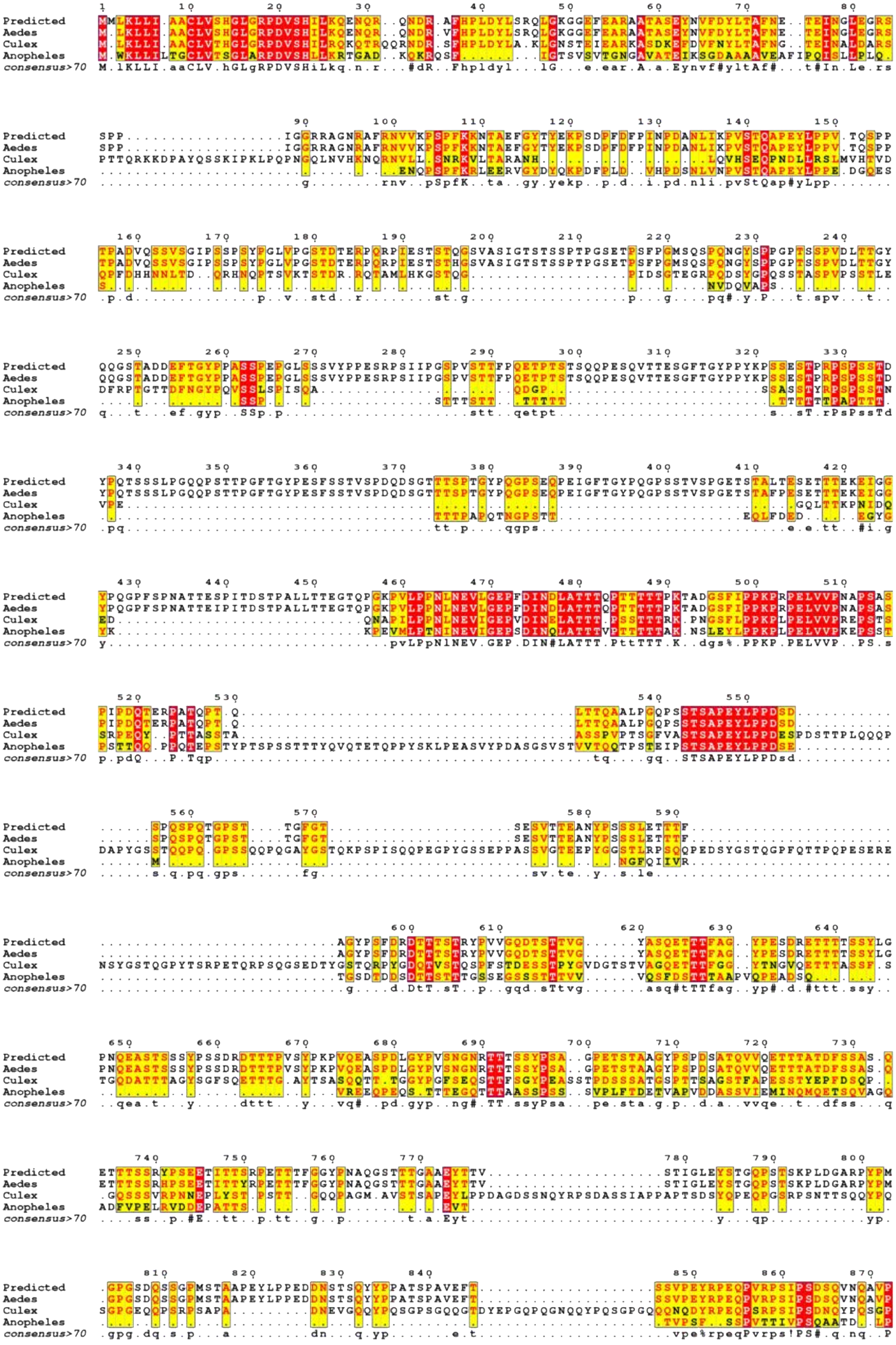

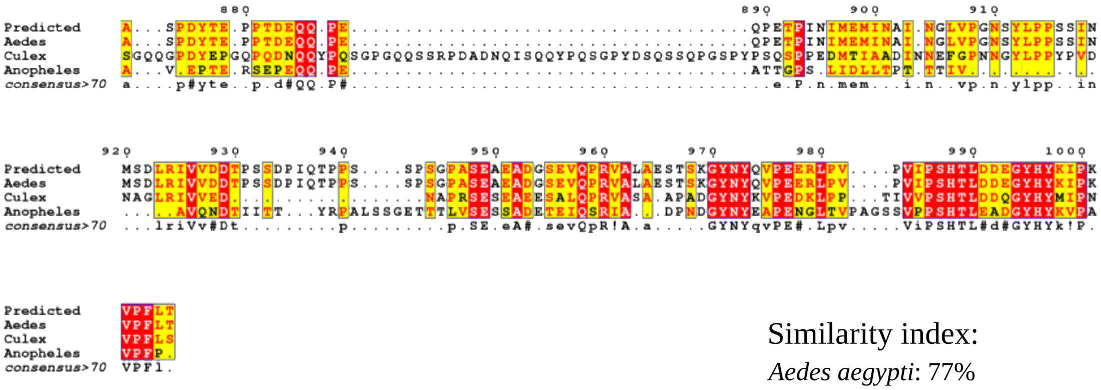

Similarity index:

Aedes aegypti: 77%

Culex quinquefasciatus: 64%

Anopheles gambiae: 56%

GALNT6

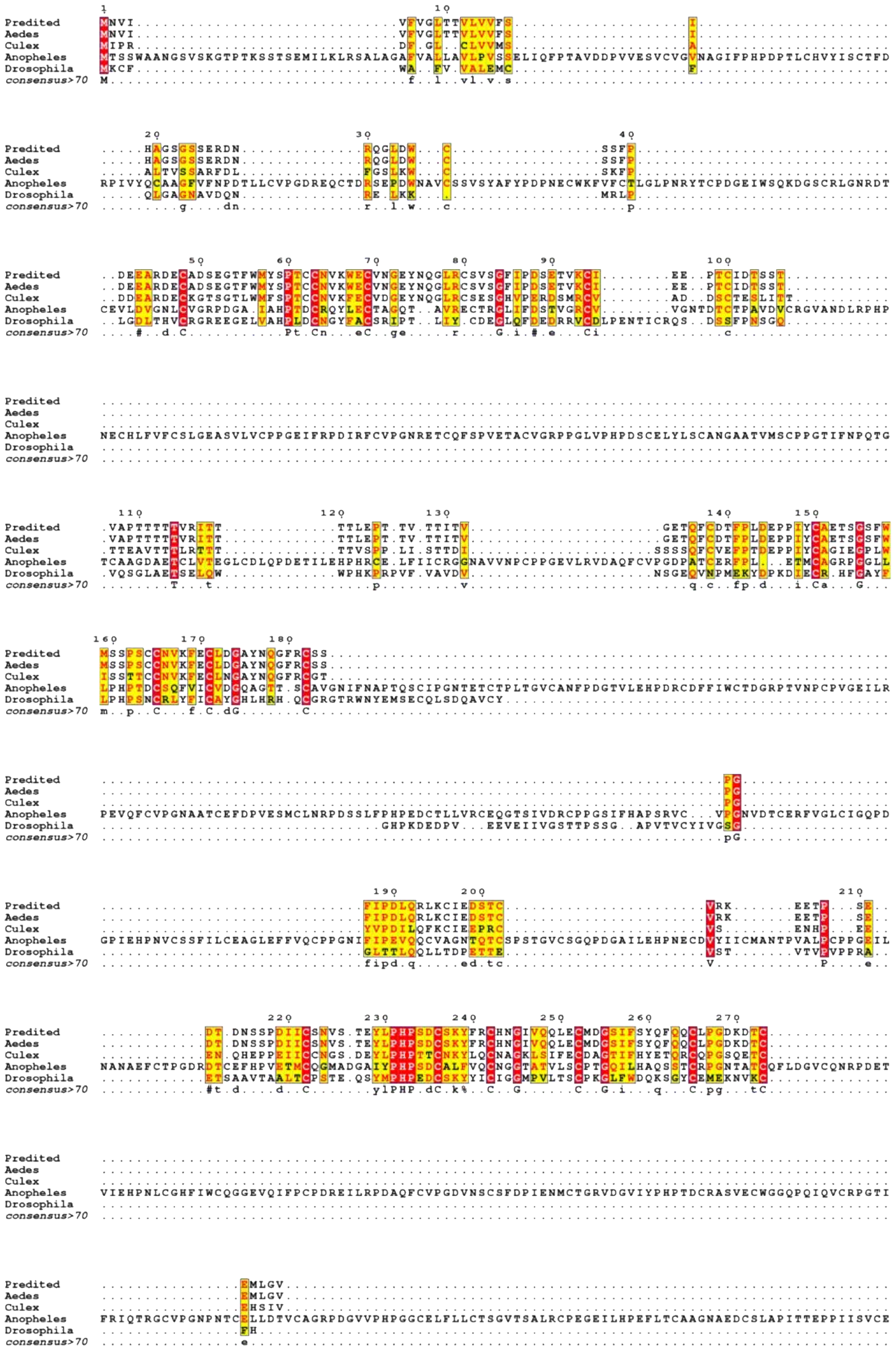

```
Predited       .....................................................
Aedes          .....................................................
Culex          ....................................YA...............
Anopheles      GRPDGNYTHPLLCYLFIRCTAGETDILSCPPNHIFVGAIRDCAPGNQETCIPF
Drosophila     ....................................................K
consensus>70   .....................................................
```

Similarity index:

Aedes aegypti: 72%
Culex quinquefasciatus: 66%
Anopheles sinensis: 56%
Drosophila ananassae: 54%

ChtBD2

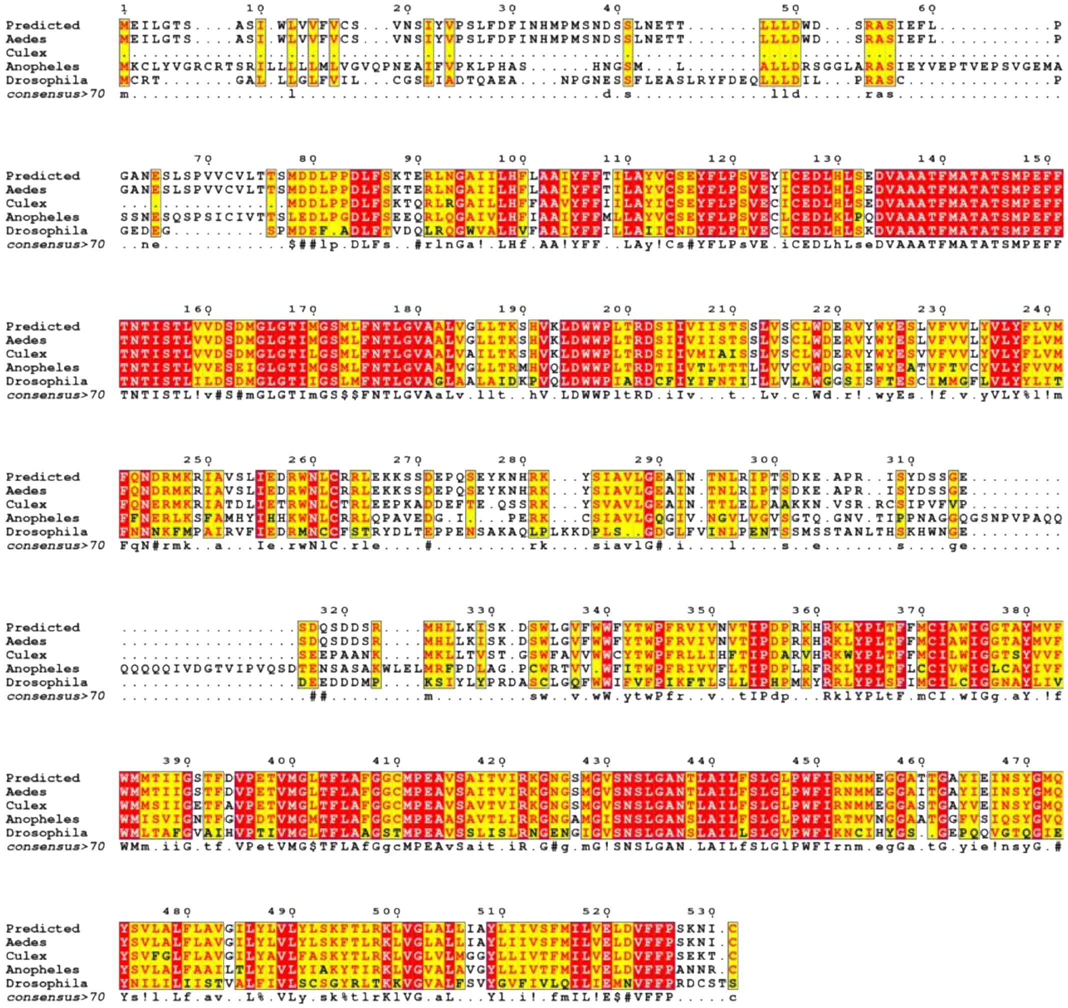

Similarity index:

Aedes aegypti: 89%
Culex quinquefasciatus: 91%
Anopheles darlingi: 85%
Drosophila serrata: 84%

KSPI

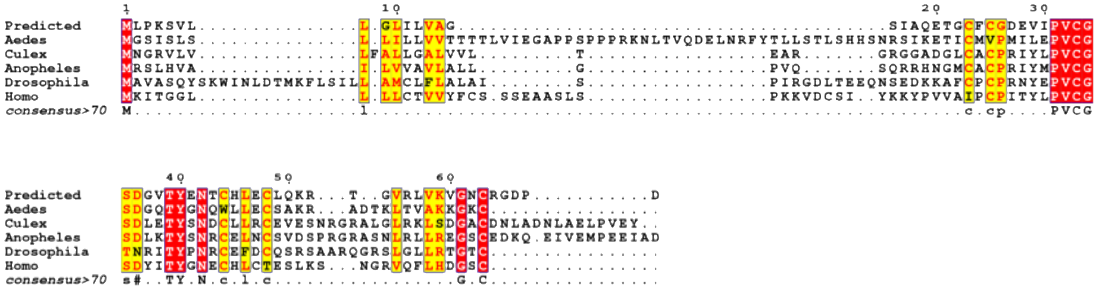

Similarity index:

Aedes aegypti: 67%

Culex pipiens pallens: 67%

Anopheles sinensis: 68%

Drosophila serrata: 67%

Homo sapiens: 61%

Na^+/Ca^{2+}

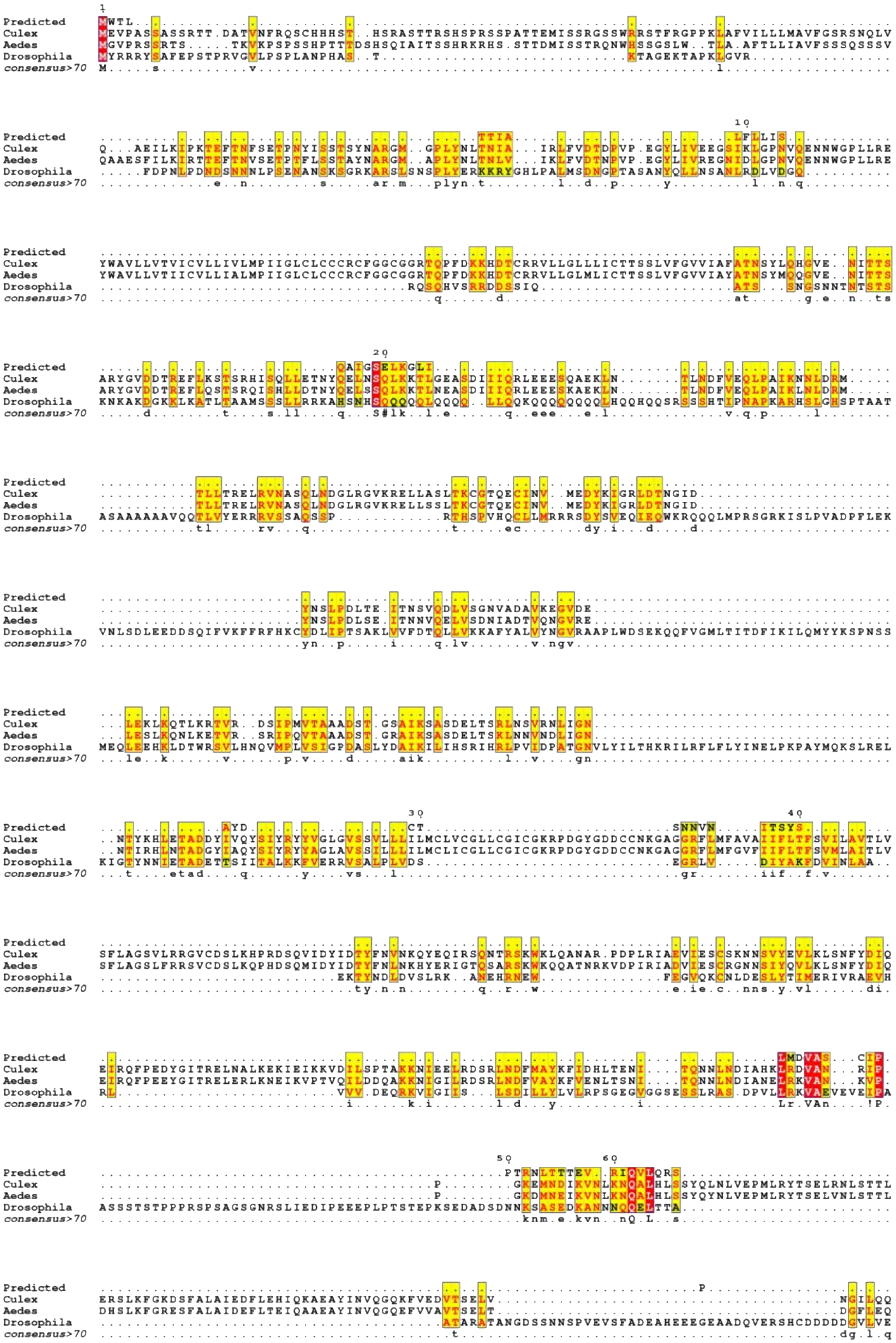

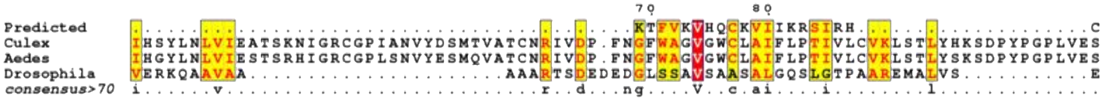

Similarity index:
Aedes aegypti: 68%
Culex quinquefasciatus: 68%
Drosophila bipectinata: 46%

Figure 2. Sequence alignment of the predicted proteins. The predicted protein sequences were compared with the homologous proteins of *Ae. aegypti*, *Culex spp.*, *Anopheles spp.*, *Drosophila spp.* and *Homo sapiens* available at NCBI database.
